# Manipulating mitochondrial reactive oxygen species alters survival in unexpected ways in a *Drosophila* Cdk5 model of neurodegeneration

**DOI:** 10.1242/bio.060515

**Published:** 2024-10-14

**Authors:** Andrew P. K. Wodrich, Brent T. Harris, Edward Giniger

**Affiliations:** ^1^ National Institutes of Health, National Institute of Neurological Disorders and Stroke, Bethesda, MD 20892, USA; ^2^ Georgetown University, Interdisciplinary Program in Neuroscience, Washington, DC 20057, USA; ^3^ University of Kentucky school of Medicine, Lexington, KY 40536, USA; ^4^ Georgetown University, Department of Pathology, Washington, DC 20057, USA; ^5^ Georgetown University, Department of Neurology, Washington, DC 20057, USA

**Keywords:** Cdk5, Mitochondria, Reactive oxygen species (ROS), Neurodegeneration

## Abstract

Reactive oxygen species (ROS) are associated with aging and neurodegeneration, but the significance of this association remains obscure. Here, using a *Drosophila* Cdk5 model of age-related neurodegeneration, we probe this relationship in the pathologically relevant tissue, the brain, by quantifying three specific mitochondrial ROS and manipulating these redox species pharmacologically. Our goal is to ask whether pathology-associated changes in redox state are detrimental for survival, whether they may be beneficial responses to pathology, or whether they are covariates of pathology that do not alter viability. We find, surprisingly, that increasing mitochondrial H_2_O_2_ correlates with improved survival. We also find evidence that drugs that alter the mitochondrial glutathione redox potential modulate survival primarily through the compensatory effects they induce rather than through their direct effects on the final mitochondrial glutathione redox potential. We also find that the response to treatment with a redox-altering drug varies depending on the age and genotype of the individual receiving the drug as well as the duration of the treatment. These data have important implications for the design and interpretation of studies investigating the effect of redox state on health and disease as well as on efforts to modify the redox state to achieve therapeutic goals.

## Teaser

**Summary:** In the context of age-related neurodegeneration in *Drosophila*, changes in three mitochondrial reactive oxygen species in the brain are related to altered survival in varied and unexpected ways.

## INTRODUCTION

Reactive oxygen species (ROS) are known to be produced in higher quantities with age and neurodegeneration, but the significance of these changes and their relationship to organismal health is unclear (Hou et al., 2019; Liu et al., 2017; Stefanatos and Sanz, 2018). Dating back to Harman's free radical theory of aging, researchers have generally thought of ROS as detrimental byproducts of metabolism (Flohé, 2020; Harman, 1956). Indeed, an imbalance of redox homeostasis favoring high levels of ROS has been shown to be detrimental in some contexts (Shields et al., 2021). However, recent studies challenge the claim that ROS are invariably harmful (Lennicke and Cochemé, 2020; Ristow and Schmeisser, 2011; Shields et al., 2021). For example, inducing ROS production has been shown to extend lifespan in multiple species (Lee et al., 2010; Schroeder et al., 2013), while manipulations of antioxidant genes have surprisingly variable effects on organismal fitness despite altered levels of ROS (Page et al., 2010; Pérez et al., 2009; Ran et al., 2007; Van Raamsdonk and Hekimi, 2009). There remains no clear-cut answer to the question of whether ROS are detrimental by-products of metabolism or potentially beneficial signaling molecules, or whether either can be true, depending on the context (Wodrich et al., 2023). It also remains unclear whether the changes in redox state that occur in aging and neurodegeneration reflect a homeostatic response, contribute to dysfunction, or simply occur in parallel with the causative pathology. Moreover, it remains uncertain how aging and pathology modify the capacity to adapt to subsequent shifts in redox state.

Uncertainties remain, in part, due to the challenges of investigating, reporting, and discussing ROS. First, the umbrella term ‘ROS’ is often used to discuss this entire class of molecules despite evidence that these molecules do not always act in concert (Lennicke and Cochemé, 2021; Sanz, 2016; Sies et al., 2022). Second, changes in a single reactive oxygen species have sometimes been viewed as reflective of a change in ROS generally (Lennicke and Cochemé, 2021; Murphy et al., 2022; Sanz, 2016). Third, certain methods of measuring ROS can be non-specific and, thus, difficult to interpret unambiguously due to molecular interactions with multiple ROS species or variable performance in differing pH environments, for example (Murphy et al., 2022; Sanz, 2016; Sies et al., 2022). Fourth, many studies that seek to investigate ROS do so by assessing proxy measures, such as lipid peroxidation, that correlate variably with changes in individual reactive oxygen species (Dotan et al., 2004; Murphy et al., 2022; Sanz, 2016).

Here, we use one *Drosophila* model of age-related neurodegeneration, cyclin dependent kinase 5 (Cdk5)-associated neurodegeneration, to investigate the above questions specifically as they relate to three different reactive oxygen species. Not only does this model have the inherent advantages of any *Drosophila* model of aging, namely the capacity for easy genetic manipulation and a short lifespan, but it also has the advantage that prior studies document the existence of transcriptional changes in mitochondrial redox-related processes that correlate with degeneration and survival (Shukla et al., 2022; Spurrier et al., 2018). This well-characterized model of age-related neurodegeneration relies upon altering the activity of the fly ortholog of a major human tau kinase, Cdk5 (Pao and Tsai, 2021). Cdk5 is a phylogenetically conserved, noncanonical cyclin dependent kinase that plays important roles in varied physiological processes such as neuronal migration and neuronal wiring during development (Chae et al., 1997; Connell-Crowley et al., 2000; 2007; Smith-Trunova et al., 2015), neuronal sub-cellular compartmentalization and organization (Klinman et al., 2017; Trunova et al., 2011; Wodrich et al., 2024), and synaptic function, including learning and memory (Seeburg et al., 2008; McLinden et al., 2012). In several contexts, Cdk5 seems to be particularly important for maintenance of homeostasis in various processes, including autophagy (Nandi et al., 2017), axon guidance (Connell-Crowley et al., 2000), and synaptic scaling (Seeburg et al., 2008). Accordingly, proper neuronal function relies upon tight regulation of Cdk5 activity, and alterations in Cdk5 activity are associated with many age-related neurodegenerative diseases, including Alzheimer disease, Parkinson disease, and amyotrophic lateral sclerosis (Nguyen et al., 2001; Patrick et al., 1999; Qu et al., 2007; Smith et al., 2003). Cdk5 activity in mammals requires binding by one of its paralogous activating subunits, either p35 or p39, that are expressed only in postmitotic neurons, thereby limiting Cdk5 activity to that cell type (Pao and Tsai, 2021). In *Drosophila*, Cdk5 has only one activating subunit, the p35 ortholog Cdk5α, and knocking out Cdk5α eliminates Cdk5 activity altogether (Connell-Crowley et al., 2007; Kissler et al., 2009). Importantly, inactivating Cdk5 by knocking out Cdk5α (Cdk5α-KO or KO) in flies causes several age-related neurodegenerative phenotypes such as shortened lifespan, axonal degeneration, impaired autophagy, motor impairment, altered innate immunity, and accelerated aging (Connell-Crowley et al., 2000; 2007; Howell et al., 2012; Kissler et al., 2009; Nandi et al., 2017; Shukla et al., 2019; Spurrier et al., 2018; 2019; Trunova and Giniger, 2012). Moreover, Cdk5α-KO flies demonstrate age-dependent degeneration of dopamine neurons and neurons in the mushroom body (MB), the region of the *Drosophila* central brain involved in learning and memory (Shukla et al., 2019; Spurrier et al., 2018; Trunova and Giniger, 2012).

Here we systematically measure three mitochondrial reactive oxygen species *in situ* in the brains of wild-type (WT) and Cdk5α-KO flies at multiple ages, both with and without administration of drugs that have well-characterized effects on ROS. We then correlate the effects of the drugs on the levels of mitochondrial ROS with their effects on organismal survival to distinguish between beneficial, detrimental, or neutral effects on the animal. In particular, we ask whether pharmacological manipulations that either reverse or exacerbate the redox changes observed in Cdk5α-KO are beneficial or detrimental to the survival of these mutant flies. We find here that Cdk5α-KO flies have a reduced mitochondrial glutathione redox potential and lower mitochondrial H_2_O_2_ level across the lifespan than controls. Furthermore, we demonstrate that the lower mitochondrial H_2_O_2_ level is likely contributing to the reduced survival in these flies, and, indeed, that there is a general correlation between higher mitochondrial H_2_O_2_ level and improved survival. In contrast, drugs that modify the mitochondrial glutathione redox potential seem to modulate survival through the compensatory effects they induce and not by their effect on the final, net level of mitochondrial glutathione oxidation. Surprisingly, we do not uncover any clear evidence for a deleterious effect of the potent oxidizing agent, superoxide, though its level does change with age in WT flies. Finally, we observe multiple contexts where chronic exposure to various drugs that target redox pathways have effects that are very different to their effects after acute administration. This suggests that compensatory metabolic rewiring occurs in disease-relevant tissues in response to redox-altering drugs in a way that is both age- and genotype-dependent.

## RESULTS

### Baseline measurements of changes in three reactive oxygen species

**Fig. 1.**
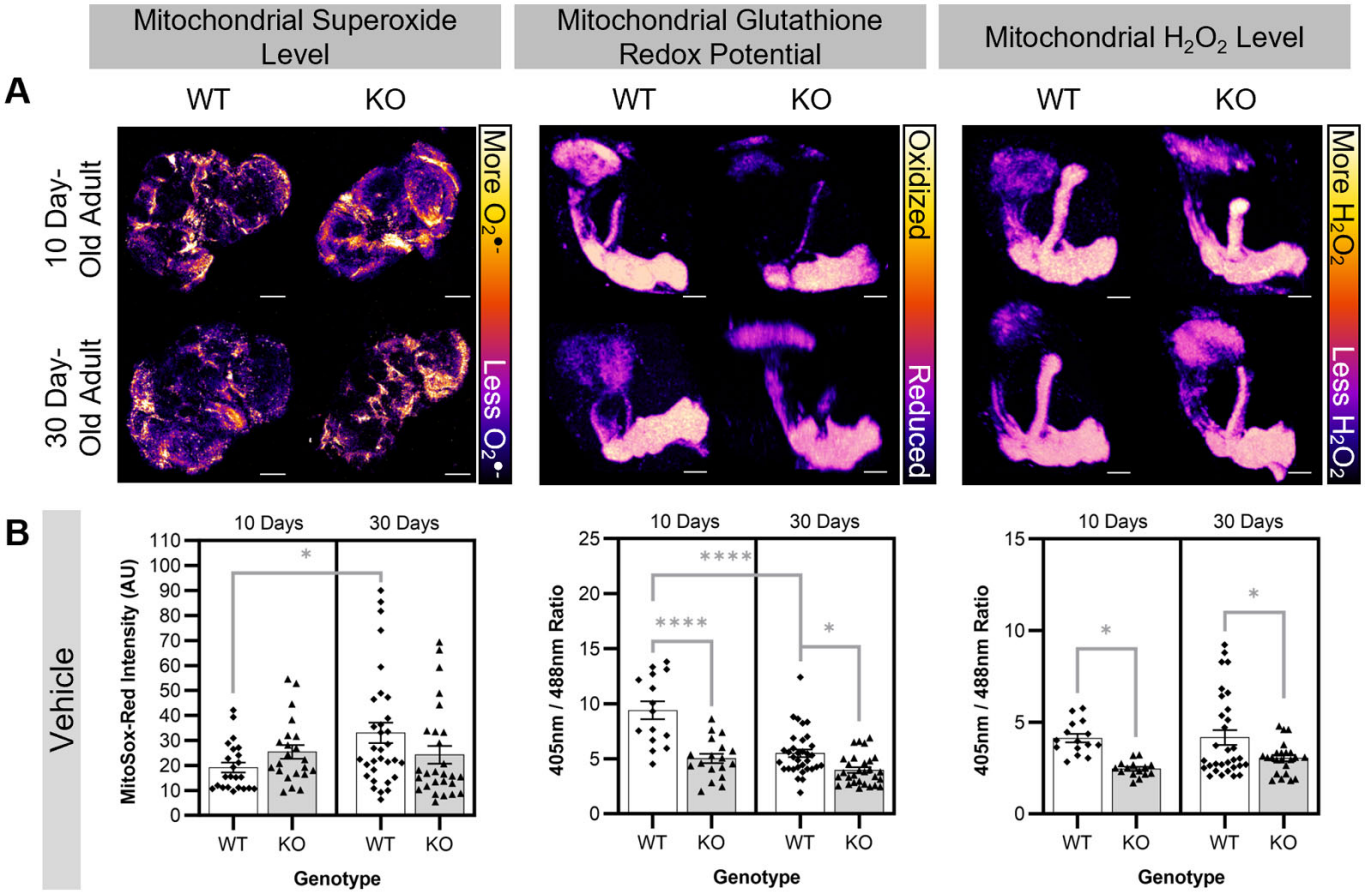
**Baseline measurements of changes in three reactive oxygen species in WT and Cdk5α-KO.** (A) Representative images of 10-day-old WT, 10-day-old Cdk5α-KO, 30-day-old WT, and 30-day-old Cdk5α-KO brains stained with MitoSox-Red, MBs expressing *201Y>mito-roGFP2-Grx1*, or MBs expressing *201Y>mito-roGFP2-Orp1* used to determine mitochondrial superoxide level, mitochondrial glutathione redox potential, or mitochondrial H_2_O_2_ level, respectively. For the mitochondrial superoxide level images, the scale bars represent 100 µm. For the mitochondrial glutathione redox potential and H_2_O_2_ level images, the scale bars represent 30 µm. For all images, the ‘Fire’ look-up table was used with warmer colors (yellows and oranges) corresponding to higher ROS level or more oxidized glutathione redox potential and cooler colors (blues and purples) corresponding to lower ROS level or more reduced glutathione redox potential. (B) Quantification of mitochondrial superoxide level, mitochondrial glutathione redox potential, or mitochondrial H_2_O_2_ level in WT or Cdk5α-KO flies fed vehicle chronically until aged 10-days old or 30-days old (DMSO or EtOH; to permit comparisons with pharmacological challenge, described below). Note that there are no significant differences between the two vehicles (Fig. S4). A higher MitoSox-Red intensity indicates a higher mitochondrial superoxide level. A larger 405 nm/488 nm ratio indicates a more oxidized mitochondrial glutathione redox potential (for *201Y>mito-roGFP2-Grx1*) or a higher mitochondrial H_2_O_2_ level (for *201Y>mito-roGFP2-Orp1*). Groups were compared using two-way ANOVA with Šidák's multiple comparison test. *P* values: *<0.05; **<0.01; ***<0.001; ****<0.0001. *N*=23 (WT, 10 days old, mitochondrial superoxide level), 22 (KO, 10 days old, mitochondrial superoxide level), 32 (WT, 30 days old, mitochondrial superoxide level), 27 (KO, 30 days old, mitochondrial superoxide level), 15 (WT, 10 days old, mitochondrial glutathione redox potential), 18 (KO, 10 days old, mitochondrial glutathione redox potential), 33 (WT, 30 days old, mitochondrial glutathione redox potential), 29 (KO, 30 days old, mitochondrial glutathione redox potential), 15 (WT, 10 days old, mitochondrial H_2_O_2_ level), 15 (KO, 10 days old, mitochondrial H_2_O_2_ level), 31 (WT, 30 days old, mitochondrial H_2_O_2_ level), 23 (KO, 30 days old, mitochondrial H_2_O_2_ level).

To determine if the mitochondrial redox state is perturbed in aging and Cdk5 pathology, we used a series of reporters and dyes to assess three reactive oxygen species *in situ* in a tissue that is known to be affected by Cdk5 pathology, namely the post-mitotic neurons of the brain and, specifically, the MB (Spurrier et al., 2018). We first used MitoSox-Red, a dihydroethidium-derived probe that is widely used as a reporter for mitochondrial superoxide (Hung et al., 2018; Mukhopadhyay et al., 2007; Robinson et al., 2006; Scialò et al., 2016), to investigate changes in mitochondrial superoxide levels within the whole brain. Control experiments demonstrate that the mitochondrial superoxide levels specifically in the MB correlate closely with those in the whole brain (Fig. S1). At 10 days old, a young age when both WT and Cdk5α-KO flies are healthy, there is no difference in mitochondrial superoxide within the brain (Fig. 1). Over time, we observe an age-dependent increase in mitochondrial superoxide in WT brains similar to reports elsewhere (Scialò et al., 2016); however, this age-dependent increase fails to occur in Cdk5α-KO (Fig. 1). The failure to observe increased superoxide in Cdk5α-KO is not attributable to a reduction in the amount of mitochondria in this condition as the relative mtDNA copy number is not significantly different in WT versus Cdk5α-KO fly heads at 10 days old or 30 days old (Fig. S2). To determine if aging or Cdk5 pathology alter the mitochondrial glutathione redox potential or H_2_O_2_ level, we used *201Y-GAL4* to express mitochondrially-localized redox biosensors (*UAS-mito-roGFP2-Grx1* or *UAS-mito-roGFP2-Orp1*, respectively) in the *Drosophila* MB, a region that undergoes age-dependent neurodegeneration (Albrecht et al., 2011; Gutscher et al., 2008, 2009; Spurrier et al., 2018). These biosensors have been used previously in other tissues in *Drosophila*, and we have validated that they also work reliably within the *Drosophila* MB (Fig. S3; Albrecht et al., 2011). In both 10-day-old and 30-day-old Cdk5α-KO adults, there is a reduction in the mitochondrial glutathione redox potential and lower mitochondrial H_2_O_2_ level relative to WT (Fig. 1). Importantly, these findings are not attributable to changes in mitochondrial mass or neuron number in the MB since these biosensors are ratiometric and, therefore, intrinsically normalized for tissue mass and probe expression level. Note that all groups were chronically fed a vehicle (either DMSO or EtOH) to permit comparisons with pharmacological challenge, described below (Table S1). Control experiments indicate that there are no differences in survival, mitochondrial superoxide level, mitochondrial glutathione redox potential, nor mitochondrial H_2_O_2_ level between the two vehicles used, and, thus, vehicle data have been combined in all experiments (Fig. S4). We note that there is an effect of the vehicle treatment specifically on the mitochondrial glutathione redox potential as compared to flies fed standard *Drosophila* media without vehicle; however, this does not affect the interpretations of the results presented here as all drug-feeding data are compared to vehicle-treated controls (Fig. S5A-C). Last, in line with previous transcriptomic data suggesting that Cdk5 pathology is associated specifically with changes in the expression of genes implicated in ROS-related processes in mitochondria, there do not appear to be global changes in ROS outside the mitochondria as we do not detect changes in the level of non-specific cytosolic ROS in the brain with age or Cdk5 pathology as measured by H_2_DCFDA, a fluorescein-derived, cytosol-localized probe that interacts with a variety of ROS (Fig. S5D; Kalyanaraman et al., 2012; Spurrier et al., 2018).

### Acute feeding of redox-altering drugs

**Fig. 2.**
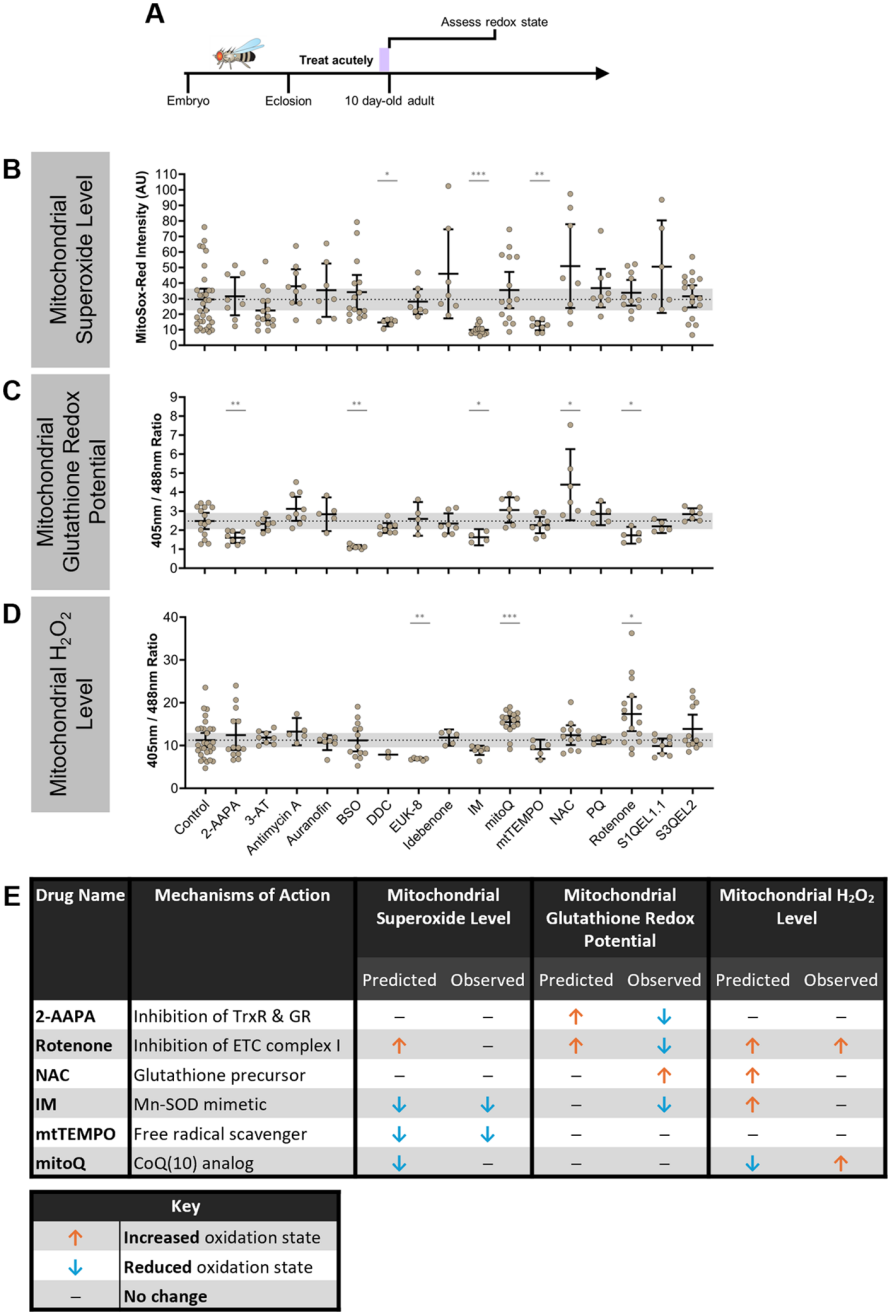
**Acute feeding of redox-altering drugs.** (A) Schematic of experimental design. WT flies were aged until 9 days old before feeding drugs acutely for 1 day. At 10 days old, flies were assessed for changes in mitochondrial redox parameters. In panels (B-D), error bars represent the 95% confidence intervals, and the gray shaded background represents the 95% confidence interval of controls. Differences between drug and control were compared using Kolmogorov–Smirnov tests. *P* values: *<0.05; **<0.01; ***<0.001; ****<0.0001. (B) Quantification of mitochondrial superoxide level after acute feeding with 16 different drugs or vehicle control. Note that raw MitoSox-Red values cannot be directly compared to chronic feeding experiments due to differences in imaging settings. A higher MitoSox-Red intensity indicates a higher mitochondrial superoxide level. *N*=32 (control), 8 (2-AAPA), 16 (3-AT), 9 (antimycin A), 7 (auranofin), 15 (BSO), 6 (DDC), 8 (EUK-8), 7 (idebenone), 15 (IM), 15 (mitoQ), 8 (mtTEMPO), 8 (NAC), 9 (PQ), 11 (rotenone), 6 (S1QEL1.1), 17 (S3QEL2). (C) Quantification of mitochondrial glutathione redox potential after acute feeding with 16 different drugs or vehicle control. Note that raw 405 nm/488 nm values cannot be directly compared to chronic feeding experiments due to differences in imaging settings. A larger 405 nm/488 nm ratio indicates a more oxidized mitochondrial glutathione redox potential. *N*=15 (control), 8 (2-AAPA), 7 (3-AT), 9 (antimycin A), 5 (auranofin), 7 (BSO), 8 (DDC), 5 (EUK-8), 7 (idebenone), 4 (IM), 7 (mitoQ), 8 (mtTEMPO), 6 (NAC), 5 (PQ), 5 (rotenone), 5 (S1QEL1.1), 7 (S3QEL2). (D) Quantification of mitochondrial H_2_O_2_ level after acute feeding with 16 different drugs or vehicle control. Note that raw 405 nm/488 nm values cannot be directly compared to chronic feeding experiments due to differences in imaging settings. A larger 405 nm/488 nm ratio indicates a higher mitochondrial H_2_O_2_ level. *N*=29 (control), 13 (2-AAPA), 8 (3-AT), 5 (antimycin A), 7 (auranofin), 13 (BSO), 2 (DDC), 6 (EUK-8), 5 (idebenone), 7 (IM), 16 (mitoQ), 5 (mtTEMPO), 11 (NAC), 5 (PQ), 16 (rotenone), 8 (S1QEL1.1), 12 (S3QEL2). (E) Summary table of predicted and observed effects of drugs selected for further analysis in this study. The drug effects are indicated by the orange up arrow (drug predicted to lead to an increased oxidation state), the blue down arrow (drug predicted to lead to a decreased oxidation state), or a black bar (drug predicted to led to no change in survival or oxidation state or a prediction cannot be made for the given drug).

To determine how the observed effects of aging and Cdk5α-KO on the three reactive oxygen species we have measured are related to organismal survival, we first needed the capability to reliably manipulate these redox parameters. We took a pharmacological approach to manipulating the mitochondrial redox state to have precise control over the timing and duration of manipulation and to avoid any potential disruption of development. We examined the literature to find drugs and dosages that have been reported to affect various redox parameters without inducing toxicity when fed to *Drosophila* (Table S2). We then performed a screen of 16 drugs predicted to affect one or more reactive oxygen species, based on their mechanisms of action as well as experimental evidence *in vitro* and *in vivo* (Tables S1, S2). To quantify the acute effects of these drugs, we fed them to 9-day-old WT adults for 24 h before assessing the mitochondrial superoxide level, glutathione redox potential, and H_2_O_2_ level at 10 days old, as described above (Fig. 2A). We found nine drugs that reliably alter the mitochondrial superoxide level, mitochondrial glutathione redox potential, or mitochondrial H_2_O_2_ level, or a combination thereof (Fig. 2B-D). We then selected six of these drugs based on their observed acute effects on these redox parameters to use for subsequent experiments (Fig. 2E). Note that because of differences in equipment settings, the raw values from these acute treatment experiments cannot be compared directly with the data from subsequent experiments, presented below, rather, raw data values should only be compared within each experiment.

### Chronic feeding of redox-altering drugs

**Fig. 3.**
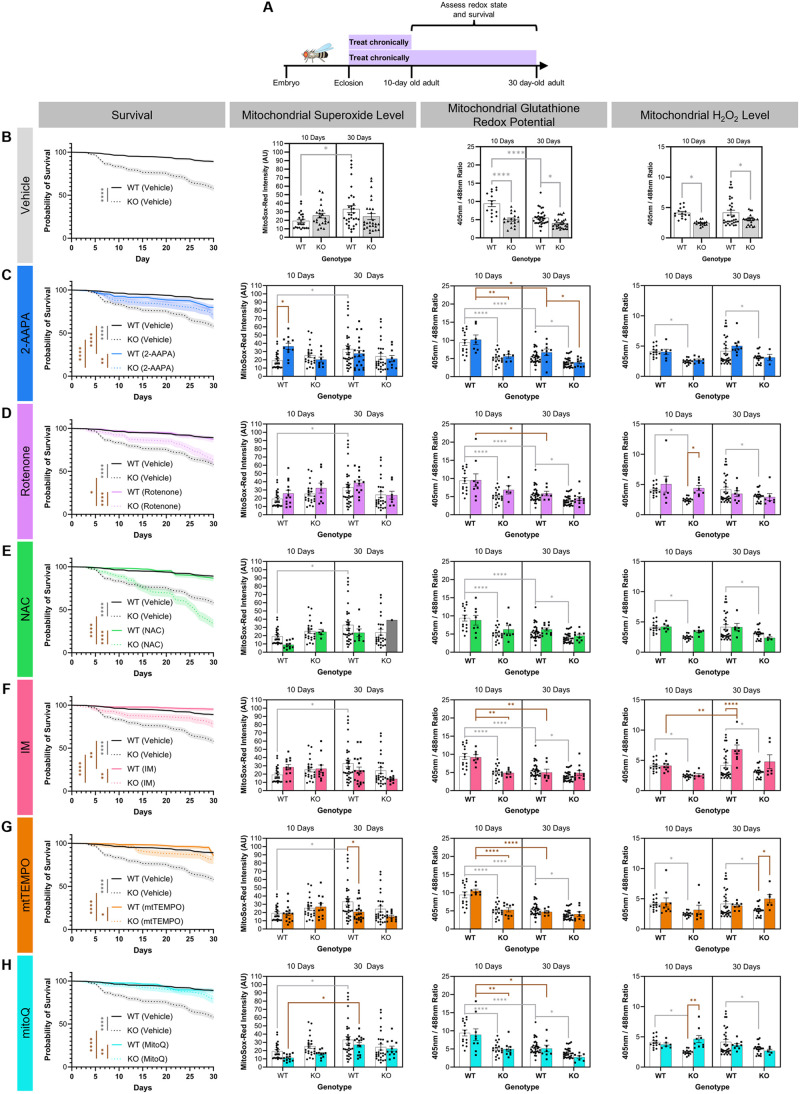
**Chronic feeding of redox-altering drugs.** (A) Schematic of experimental design. WT and Cdk5α-KO flies were chronically fed drugs from 0 days old until either 10 days old or 30 days old at which point flies were assessed for changes in mitochondrial redox parameters. Survival of all flies was monitored for 30 days. (B) Quantification of survival, mitochondrial superoxide level, mitochondrial glutathione redox potential, and mitochondrial H_2_O_2_ level in WT and Cdk5α-KO flies fed vehicle (DMSO or EtOH) only; there are no significant differences based on the vehicle used (Fig. S4). Note that the mitochondrial superoxide level, mitochondrial glutathione redox potential, and mitochondrial H_2_O_2_ level data in this panel are replotted here from Fig. 1B for the reader's convenience and all vehicle data are pooled in all the following panels of this figure to compare the effects of drug versus vehicle. Note also that the raw MitoSox-Red intensity and 405 nm/488 nm values cannot be compared directly to those from the acute drug feeding due to equipment differences. A higher MitoSox-Red intensity indicates a higher mitochondrial superoxide level. A larger 405 nm/488 nm ratio indicates a more oxidized mitochondrial glutathione redox potential (for *201Y>mito-roGFP2-Grx1*) or a higher mitochondrial H_2_O_2_ level (for *201Y>mito-roGFP2-Orp1*). Shaded backgrounds in survival figures represent s.e.m. Differences in survival among groups were assessed by Mantel–Cox log-rank test with Bonferroni multiple comparison test. Differences in mitochondrial ROS among groups were assessed by two-way ANOVA with Bonferroni multiple comparison test. Light gray significance bars represent significant comparisons between vehicle groups and are presented in all the following panels. *P* values: *<0.05; **<0.01; ***<0.001; ****<0.0001. For survival data, *N*=1482 (WT, vehicle, survival), 1022 (KO, vehicle, survival). For mitochondrial superoxide level, mitochondrial glutathione redox potential, and mitochondrial H_2_O_2_ level data, sample sizes are listed in the legend to Fig. 1B. (C-H) Quantification of survival, mitochondrial superoxide level, mitochondrial glutathione redox potential, and mitochondrial H_2_O_2_ level in WT and Cdk5α-KO flies fed 2-AAPA (C), rotenone (D), NAC (E), IM (F), mtTEMPO (G), or mitoQ (H). Note that vehicle controls are the same as shown in panel (B) and are shared for all drugs. Note that for the mitochondrial superoxide level measurement, *N*=1 for 30-day-old Cdk5α-KO, and this is denoted by a gray bar (E). A higher MitoSox-Red intensity indicates a higher mitochondrial superoxide level. A larger 405 nm/488 nm ratio indicates a more oxidized mitochondrial glutathione redox potential (for *201Y>mito-roGFP2-Grx1*) or a higher mitochondrial H_2_O_2_ level (for *201Y>mito-roGFP2-Orp1*). Shaded backgrounds in survival figures represent s.e.m. Differences in survival among groups were assessed by Mantel–Cox log-rank test with Bonferroni multiple comparison test. Differences in mitochondrial ROS among groups were assessed by two-way ANOVA with Bonferroni multiple comparison test. Brown significance bars represent significant comparisons that include drug-treated groups. *P* values: *<0.05; **<0.01; ***<0.001; ****<0.0001. Samples sizes for data in Fig. 3C-H can be found in Table S3.

To determine whether the observed age- and genotype-dependent changes in three reactive oxygen species contribute to organismal decline or are a homeostatic response to limit decline, we used the drugs identified above at the same dosages to alter the mitochondrial redox state in WT and Cdk5α-KO across the lifespan. We then assessed how rescuing or exacerbating the changes in ROS that we describe above affects survival in these flies. For this chronic feeding, we fed drugs to WT or Cdk5α-KO flies from the day they eclosed as adults until they were 10 days old or 30 days old, at which point we assessed mitochondrial superoxide level, glutathione redox potential, and H_2_O_2_ level (Fig. 3A). We also assessed survival in response to drug treatment in both WT and Cdk5α-KO flies until they were 30 days old (Fig. 3A). Importantly, the addition of redox altering drugs to the fly food does not alter the uptake of food relative to vehicle control [when assayed in sucrose media (Fig. S6) though formally we cannot exclude the possibility of an effect of drug on feeding preference in other food formulations]. Note that we were unable to reliably assess the effect of drug treatment on neuron cell loss in the MB since various drug treatments interfered with the methods we used previously to quantify neuron number (discussed in Materials and Methods). We found several changes in mitochondrial redox parameters and survival in response to redox-altering drug treatments. In this study, we attribute the survival effects of Cdk5α-KO to its effects in the nervous system since that is the only tissue where significant Cdk5α expression and Cdk5 activity have been reported in the fly, and we use the redox state of the MB as a proxy for Cdk5-sensitive neurons overall since it is the region of the fly brain where the effects of Cdk5 have been best characterized (Connell-Crowley et al., 2000; 2007; Spurrier et al., 2018; Trunova and Giniger, 2012). Moreover, the MB has previously been implicated in control of *Drosophila* lifespan (Lien et al., 2020). Here, we highlight salient observations based on examining changes across reactive oxygen species, drug, age, and genotype (Table 1).

**Table 1.**
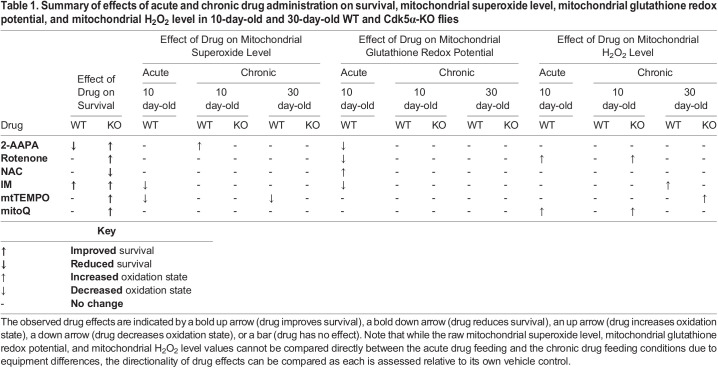
Summary of effects of acute and chronic drug administration on survival, mitochondrial superoxide level, mitochondrial glutathione redox potential, and mitochondrial H_2_O_2_ level in 10-day-old and 30-day-old WT and Cdk5α-KO flies

Looking across reactive oxygen species, we see numerous differences in how individual reactive oxygen species respond to various drug treatments. For example, none of the six drugs we assayed significantly alters the glutathione redox potential after 10 days or 30 days of chronic treatment as compared to vehicle-treated controls (Fig. 3). In contrast, rotenone and mitoQ both increase mitochondrial H_2_O_2_ in 10-day-old Cdk5α-KO adults and this restoration of mitochondrial H_2_O_2_ towards the WT baseline correlates with improvement of Cdk5α-KO survival, suggesting that the diminished mitochondrial H_2_O_2_ in Cdk5α-KO may actually be detrimental to organismal survival (Fig. 3). Consistent with this, increases in mitochondrial H_2_O_2_ in 30-day-old WT and Cdk5α-KO adults in response to IM and mtTEMPO, respectively, correlate with improvements in survival and further support a positive correlation between increased mitochondrial H_2_O_2_ and improved survival (Fig. 3).

Looking across drugs, we observe that chronic administration of 2-AAPA has a discordant effect on survival between WT and Cdk5α-KO: WT survival is worsened by treatment with 2-AAPA while Cdk5α-KO survival is improved (Fig. 3C). However, the only net change on mitochondrial ROS induced by chronic 2-AAPA is an increase in mitochondrial superoxide level in 10-day-old WT, which cannot by itself account for the differing survival effects in WT versus Cdk5α-KO (Fig. 3C). We also note that chronic administration of NAC, despite inducing oxidation of the mitochondrial glutathione redox potential after acute administration, has no effect on any of the mitochondrial redox parameters we examined after chronic administration, suggesting that drugs may exert different effects or be processed differently depending on the duration of drug administration (Fig. 3E). Lastly, we see that chronic administration of either IM or mtTEMPO induces an age-dependent change in mitochondrial H_2_O_2_ level or superoxide level, respectively; that is, neither drug induces a change in mitochondrial redox parameters after 10 days of chronic administration, but they both induce effects after 30 days of chronic administration, suggesting that either the age of the animal or the duration of drug treatment is modulating its redox effect (Fig. 3F,G).

Several of our findings, including those described above, suggest that compensatory physiological responses to some drugs may have effects that alter or limit the direct effects of those agents, as determined by acute administration of these drugs to 10-day-old WT flies (Table 1). Thus, drugs administered acutely induce numerous significant effects on mitochondrial superoxide level, glutathione redox potential, and H_2_O_2_ level in 10-day-old WT flies. However, when these same drugs are administered chronically in either WT or Cdk5α-KO, many of these effects vanish. For the current analysis, we assume that the direct effect of each drug is similar in WT and Cdk5α-KO flies, but we cannot formally exclude the possibility that the acute effect of a drug may be altered in Cdk5α-KO.

As examples of compensation to chronic drug treatment, in WT flies, acute administration of 2-AAPA, rotenone, or IM reduces the mitochondrial glutathione redox potential, and NAC oxidizes it, but none of these alters the final mitochondrial glutathione redox potential measured after chronic drug administration (Figs 2, 3). The survival consequences of these examples of compensation to drug are surprisingly varied, however, as IM improves survival, NAC worsens survival only in Cdk5α-KO, rotenone improves survival only in Cdk5α-KO, and 2-AAPA causes opposing survival effects in WT versus Cdk5α-KO (Fig. 3; in the Discussion, we will consider how these seemingly inconsistent results may be reconciled). Genotype has yet other consequences for the ability to compensate for drug treatment, moreover. We observe, for example, that Cdk5α-KO flies show changes in mitochondrial H_2_O_2_ level under chronic treatment with rotenone or mitoQ that match the direct effects of these drugs observed upon acute treatment; that is, the mutant flies fail to compensate for the acute effects of these drugs on mitochondrial H_2_O_2_. In contrast, WT flies completely suppress the direct effect of these drugs in the same chronic treatment condition and show no change in final mitochondrial H_2_O_2_ (Figs 2 and 3). This could imply that Cdk5α-KO impairs the homeostatic machinery that would normally compensate for the effects of drugs, or that the baseline redox state of Cdk5α-KO is intrinsically more sensitive to the effects of drugs. Acute administration of IM and mtTEMPO each causes a decrease in mitochondrial superoxide level while chronic administration of either drug for 10 days does not affect mitochondrial superoxide level in WT, suggesting that there is a compensatory response to these drugs that limits their effect on this redox parameter (Figs 2 and 3). Furthermore, the effect of the drug, the compensatory response to the drug, or a combination of these prevents the age-dependent increase in mitochondrial superoxide level that occurs without drug in WT (Fig. 3). However, the consequences these effects on mitochondrial superoxide may have for survival cannot be determined from these data, as chronic administration of either IM or mtTEMPO also increases the mitochondrial H_2_O_2_ level which we have already found to be strongly associated with improved survival, as discussed above. We also note that we cannot exclude the possibility that some portion of the differences in response to drug observed in acute versus chronic experiments may have been affected by complex interactions between the drug and the feeding media itself, which was sucrose in the acute experiments but *Drosophila* media in the chronic experiments.

### Effects of redox-altering drug treatment duration versus age at treatment assessment

**Fig. 4.**
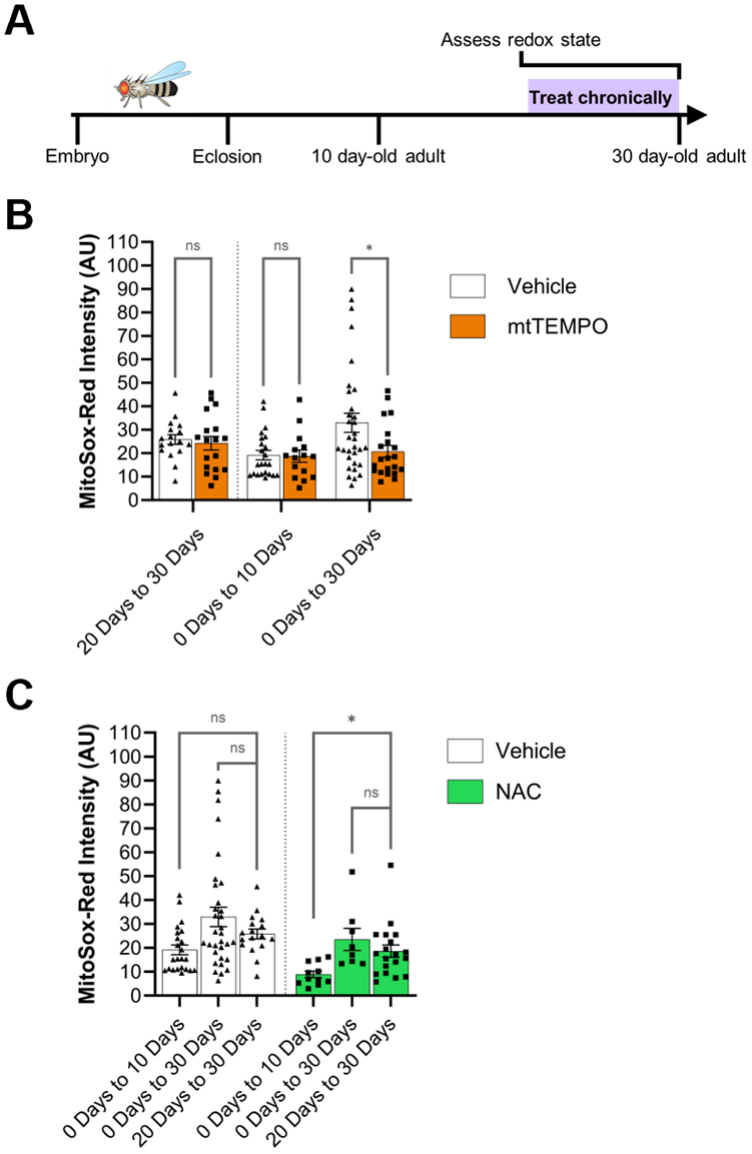
**Effects of redox-altering drug treatment duration versus age at treatment assessment.** (A) Schematic of experimental design. WT flies were raised until 20 days old without drug treatment before being fed drugs chronically until 30 days old. The mitochondrial superoxide level was then assessed in these flies at 30 days old and compared to data from Fig. 3. (B) Comparison of mitochondrial superoxide level in flies fed mtTEMPO versus vehicle from 20 days old until 30 days old. Data from quantification of mitochondrial superoxide level of flies fed mtTEMPO from 0 days old until either 10 days old or 30 days old are the same as that presented in Fig. 3. Vehicle-treated groups were compared to mtTEMPO-treated groups for each chronic feeding paradigm by two-way ANOVA with Šidák's multiple comparison test. *P* values: *<0.05; **<0.01; ***<0.001; ****<0.0001. *N*=17 (vehicle, 20 days to 30 days), 18 (mtTEMPO, 20 days to 30 days), 23 (vehicle, 0 days to 10 days), 15 (mtTEMPO, 0 days to 10 days), 32 (vehicle, 0 days to 30 days), 21 (mtTEMPO, 0 days to 30 days). (C) Comparison of mitochondrial superoxide level among flies fed NAC or among flies fed vehicle from 20 days old until 30 days old. Data from quantification of mitochondrial superoxide level of flies fed NAC from 0 days old until either 10 days old or 30 days old are the same as that presented in Fig. 3. Vehicle-treated groups and NAC-treated groups were compared using one-way ANOVAs with Dunnett's multiple comparison tests. *P* values: *<0.05; **<0.01; ***<0.001; ****<0.0001. *N*=23 (vehicle, 0 days to 10 days), 32 (vehicle, 0 days to 30 days), 17 (vehicle, 20 days to 30 days), 11 (NAC, 0 days to 10 days), 8 (NAC, 0 days to 30 days), 19 (NAC, 20 days to 30 days).

Differences in the effects of a chronic drug treatment on 10 day-old versus 30 day-old adults could be due to the absolute age of the animal at the time of assessment or the duration of drug treatment. Therefore, we tested whether the duration of drug treatment or the age at which the drug effect is assessed is more relevant for the response to the drug in 30-day-old adults. We compared mitochondrial superoxide levels in WT adults fed mtTEMPO, NAC, or vehicle either from eclosion to 10 days old, from eclosion to 30 days old, or for a 10-day period from 20 days old until 30 days old (Fig. 4A). Interestingly, we found evidence for each of the possible explanations, depending on the drug. For each drug, we compared directly the effect of drug versus vehicle for each treatment protocol as well as comparing across treatment protocols, both for drug and for vehicle; for simplicity, we display only the most informative comparisons here for each drug. For mtTEMPO, which shows a significant effect on mitochondrial superoxide level with treatment from 0 days to 30 days as compared to vehicle-treated controls, we find that feeding drug to WT flies just from 20 days old to 30 days old does not alter the mitochondrial superoxide level as compared to control, which matches the effect of mtTEMPO feeding from eclosion until 10 days old (Fig. 4B; Fig. S7A). This suggests that the duration of treatment is more relevant to the effect of this drug than the age of the animal at the time of assessment. In contrast, the results of feeding WT flies NAC from 20 days old until 30 days old match closely those of feeding NAC to WT flies from 0 days old until 30 days old and are significantly different from those of feeding NAC to WT flies from 0 days old until 10 days old, arguing that actual age at the time of assessment is more important for the observed effects of the drug rather than the duration of treatment (Fig. 4C; Fig. S7B). In total, this suggests that both treatment duration and age at assay can contribute to the effect of a drug on aged flies, with the dominant factor depending on the specific pharmacologic agent.

### Comparative transcriptomics of acute versus chronic redox-altering drug treatment

**Fig. 5.**
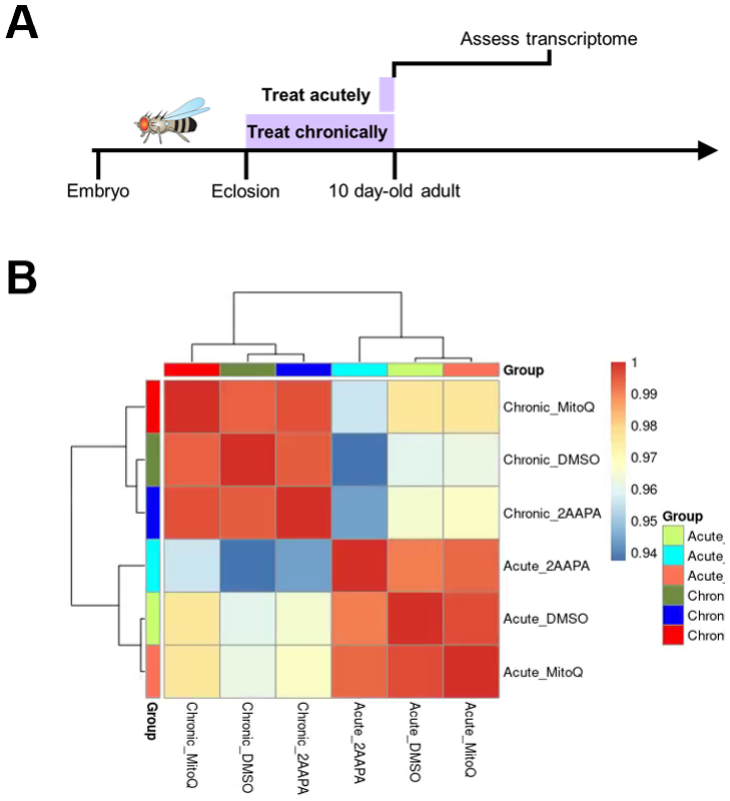
**Comparative transcriptomics of acute versus chronic redox-altering drug treatment.** (A) Schematic of experimental design. WT flies were fed drugs either acutely (for 24 h, with assay at 10 days old) or chronically (for 10 days) before brains were dissected and groups of 20 brains were pooled for transcriptomic analysis. (B) Heatmap of correlation amongst experimental groups based on 184 differentially expressed genes that contribute significantly to the variance amongst samples. Selected genes had a linear fold change of means>|1.5| and a corrected *P*<0.05. *N*=7 (acute, DMSO), 6 (acute, 2-AAPA), 7 (acute, mitoQ), 6 (chronic, DMSO), 7 (chronic, 2-AAPA), 6 (chronic, mitoQ).

It is apparent that not only the redox state, but the compensatory metabolic response to a perturbed redox state is important for survival. Thus, we chose to investigate potential mechanisms of this compensatory response. We hypothesized that compensation could be occurring through changes at the level of gene expression. We therefore selected two drugs that showed evidence of chronic compensation, 2-AAPA and mitoQ, isolated brains from 10-day-old WT animals that had been treated with these drugs either acutely or chronically, and performed bulk RNAseq analysis (Fig. 5A). Transcriptomic profiling of these flies revealed small but statistically significant differences in gene expression between the acute and chronic administration conditions, but these were attributable to effects of vehicle treatment as ANCOVA testing correcting for drug treatment duration (acute versus chronic drug treatment) reveals no differentially expressed genes among treatment conditions (vehicle versus 2-AAPA versus mitoQ; Fig. 5B; Fig. S8, Table S5). We did not observe changes that correlate with the identity of the specific drug (p>0.05 for all genes after correction for multiple testing). This argues against the hypothesis that compensation to these specific chronic drug treatments occurs at the level of transcription, suggesting instead that it is likely to occur post-transcriptionally.

## DISCUSSION

Changes in redox state have been linked to aging and to many neurodegenerative conditions (Hou et al., 2019; Liu et al., 2017; Stefanatos and Sanz, 2018). It has been confusing, however, to understand the nature of this linkage. Are the redox changes seen in different conditions responsible for organismal decline in those circumstances, are they homeostatic changes to counteract pathology, or are they simply changes occurring in parallel? Are ROS purely detrimental metabolic by-products, or could they potentially be beneficial molecules? Do aging or pathology affect the ability of the redox metabolic environment to respond to further changes in the redox state? Here, we find that the answers to these questions are nuanced, varying with the reactive oxygen species and the circumstances, yet we can extract some important conclusions from our data. We find evidence from multiple conditions that one classic reactive oxygen species, H_2_O_2_, can be beneficial for survival. On the other hand, the survival effect of pharmacologically altering a different species, glutathione, seems to be mediated primarily by the compensatory mechanisms induced by those drugs and not by their direct effects on the ROS targets we have investigated. We also find that the response to treatment with a redox-altering drug, both in terms of the direct redox response and its impact on survival, vary dramatically depending on the age at which the drug is administered, the duration of the treatment, and the genotype of the individual receiving the drug.

To disentangle the influences of genetics and pharmacology on redox state and organismal viability, we proceeded in three steps. First, to define a baseline, we quantified three reactive oxygen species at multiple ages in the brains of WT flies and those with a genetic mutation, Cdk5α-KO, which disrupts the redox state, shortens lifespan, and induces adult-onset neurodegeneration. Second, we identified a set of drugs that alter mitochondrial redox parameters in the WT brain in well-characterized ways when administered acutely. Finally, we assessed whether the alterations in redox state produced by Cdk5α-KO are causal for reduced lifespan by testing whether drugs that tend to reverse Cdk5α-KO-dependent mitochondrial redox changes also tend to improve viability and whether drugs that exacerbate the redox effects of Cdk5α-KO tend to further reduce survival. In this study, we assayed ROS specifically in the brain because Cdk5 is active only in neural tissue, and we focused our attention on the MB as it is a well-documented target of Cdk5 action (Connell-Crowley et al., 2000; 2007; Spurrier et al., 2018; Trunova and Giniger, 2012). Importantly, changes in survival in Cdk5α-KO are attributable to changes in the Cdk5-sensitive tissue, the post-mitotic neurons, and the MB has been shown previously to regulate lifespan in the fly (Lien et al., 2020), making it likely that the mitochondrial redox changes we assay in the MB are related directly or indirectly to those changes in survival.

In Cdk5α-KO, we made the unexpected observation that the level of mitochondrial H_2_O_2_ actually diminishes with age in a Cdk5-sensitive region of the brain, the MB. To test whether this decrease in mitochondrial H_2_O_2_ level is itself detrimental to survival or is a homeostatic response to pathology, we treated Cdk5α-KO flies with either of two drugs, rotenone and mitoQ, that raise the level of mitochondrial H_2_O_2_, and in each case we found that the drug treatment improves viability. This suggests that increased mitochondrial H_2_O_2_ level, rather than being detrimental to survival, may actually be beneficial. Further tests reinforce this conclusion as raising the mitochondrial H_2_O_2_ level in WT, by chronic treatment with IM, also improves survival. The observation that increasing mitochondrial H_2_O_2_ has beneficial consequences for survival in multiple conditions runs counter to the notion that increases in ROS are necessarily detrimental. In this connection, it is interesting that a previous study also found that decreased mitochondrial H_2_O_2_ level (due to physical activity) was associated with a decrease in lifespan in *Drosophila*, though that seemingly paradoxical observation was not investigated further (Cochemé et al., 2011). It has been argued that protective effects of drugs that increase ROS levels are an example of hormesis, wherein a temporary increase of biological stress induces long-term survival benefits through the activation of genetic programs that counteract the effect (Bárcena et al., 2018; Cheng et al., 2023; Ristow and Zarse, 2010; Tapia, 2006). However, if this were the case, we should observe a long-term reduction in mitochondrial H_2_O_2_ level in the rescued condition, whereas we observe an increase. This argues against the simplest version of the hormesis model in this case, though more complex models are possible. The mechanisms by which mitochondrial H_2_O_2_ produces its pro-survival effect are unclear, though one could speculate about potential interactions with redox-sensitive signaling molecules or transcription factors (Lennicke and Cochemé, 2021).

Analyses of the changes in mitochondrial glutathione redox potential provide informative contrasts to the results concerning mitochondrial H_2_O_2_. After chronic vehicle treatment, we observed reduction of the mitochondrial glutathione redox potential in the MB of Cdk5α-KO. However, none of the four drugs that altered the mitochondrial glutathione redox potential upon acute administration – 2-AAPA, rotenone, NAC, and IM – altered glutathione redox potential relative to vehicle after chronic administration in Cdk5α-KO flies. Nonetheless, all these drugs did modify Cdk5α-KO survival relative to vehicle in ways that correlate with their acute effect on the mitochondrial glutathione redox potential. Acute administration of NAC oxidizes the mitochondrial glutathione redox potential and chronic NAC administration worsens Cdk5α-KO survival; on the contrary, acute administration of 2-AAPA, rotenone, and IM all reduce the mitochondrial glutathione redox potential and chronic administration of these drugs improves Cdk5α-KO survival. In both scenarios, however, these survival effects occur despite these drugs not altering the final mitochondrial glutathione redox potential. While we cannot rule out effects of these drugs on other ROS that we have not measured, one intriguing possibility is that the mechanisms responsible for the alteration of survival after chronic feeding with 2-AAPA, rotenone, NAC, and IM in Cdk5α-KO are the same compensatory mechanisms that prevent chronic 2-AAPA, rotenone, NAC, and IM exposure from affecting the final mitochondrial glutathione redox potential after treatment, rather than the direct effect of the drugs themselves. Interestingly, the same survival patterns do not apply upon treatment of WT flies with these drugs, perhaps suggesting genotype-dependent differences, as will be discussed further below. In total, this suggests that compensation to acute oxidation of the mitochondrial glutathione redox potential may be associated with worsened survival while compensation to acute reduction of the mitochondrial glutathione redox potential may be associated with improved survival, at least for Cdk5α-KO flies.

Our data also do not reveal obvious evidence for detrimental effects of changes in mitochondrial superoxide level. It is interesting to note that superoxide is often argued to be a particularly damaging reactive oxygen species, yet we observe an age-related increase in the brains of WT flies that is not associated with any survival effect and suppressing this age-related increase does not alter survival, which argues against the notion that mitochondrial superoxide is necessarily detrimental to survival, at least at the levels generated in this study (Shields et al., 2021).

We observe here multiple instances where a redox parameter is changed after acute exposure of a drug but not after chronic exposure to the same drug, suggesting that compensatory metabolic changes occur to alter drug uptake, metabolism or final effect. For example, acute feeding of IM or 2-AAPA to 10-day-old WT flies induces a decrease in mitochondrial superoxide level or a reduction in the mitochondrial glutathione redox potential, respectively; however, upon chronic feeding of these drugs to WT flies, these flies show neither effect. Apparently, the redox state is very malleable to acute administration of redox-altering drugs but less so after chronic administration. The simplest explanation is that there is a compensatory reprogramming of cellular metabolism in response to the drug, whether by altering its bioavailability or its downstream effects. These two possibilities cannot be distinguished unambiguously from the data here, though the occurrence of survival effects in the absence of net changes in redox state upon chronic treatment tends to favor the idea that the effects act downstream. Our transcriptomic analysis of flies fed drugs acutely or chronically indicates that this compensatory reprogramming of metabolism is likely to occur post-transcriptionally, at least for the drugs we have tested, although further investigation is required to identify the mechanisms that are responsible.

It appears that the capacity for compensation to redox-altering drugs is both age- and genotype-dependent. For example, in WT flies, mtTEMPO induces an acute decrease in mitochondrial superoxide level, but this effect disappears after chronic exposure for 10 days. However, after 30 days of chronic drug exposure, the effects on mitochondrial superoxide reemerge, suggesting that age and/or treatment duration affects the ability to compensate for the effects of these drugs. Likewise, compensation can be influenced by genotype as we observe that the increase in mitochondrial H_2_O_2_ after acute administration of rotenone and mitoQ is blocked after 10 days of chronic treatment in WT, but not in Cdk5α-KO. Additionally, we observe that compensation can have genotype-dependent effects on survival. For example, the acute effect of 2-AAPA on mitochondrial glutathione redox potential is compensated for (i.e. suppressed) when administered chronically in both genotypes, yet, interestingly, 2-AAPA decreases survival in WT and rescues survival in Cdk5α-KO. Importantly, other changes in redox parameters or compensation cannot account for these survival changes in this case, at least for the ROS we have measured. These differing effects of compensation on survival suggest that there is a difference in the capacity or character of this compensation in the context of a mutation that induces neurodegeneration, and this may partly explain why redox state changes in the context of age-related neurodegeneration are variably correlated with pathology and overall organismal health. Indeed, others have commented on the differing metabolic environments that exist in disease states and how reprogrammed metabolisms can alter responses to pharmacologic manipulations, for example (Parkhitko et al., 2020). It is also intriguing to speculate that metabolic reprogramming after mitochondrial redox-altering drug treatment may influence survival by altering other mitochondrial-based processes besides redox state that have been shown to influence lifespan across numerous species, such as oxidative phosphorylation, beta-oxidation, and the mitochondrial unfolded protein response (Bar–Ziv et al., 2020; Johnson and Stolzing, 2019; Parkhitko et al., 2020; Scialo et al., 2013; Wodrich et al., 2022). It may also be that the observation that both aged flies and Cdk5α-KO flies are less capable of compensation to redox-altering drugs is related to the previous finding that Cdk5α-KO leads to an acceleration of aging (Spurrier et al., 2018). This may suggest that aging, and degenerative pathologies that accelerate aging like Cdk5α-KO, may be less amenable to treatment with drugs that exert their beneficial effects through a rewiring of metabolism rather than a direct effect on their target. It also implies that caution must be used whenever effects of a drug tested on a young, healthy population are extrapolated to a population that is aged or infirm.

Another unexpected finding from our study was the poor correlation between the predicted effects of a drug and its effects in the brain. This may arise from the use of predictions based on the *in vivo* effects of these drugs in tissues other than the brain or from predictions derived from experiments in simplified systems, such as measurements of ROS obtained from isolated mitochondria that lack the interactions found amongst native cells and tissues (Sanz, 2016). The discordance between the predicted effect of a drug and observed effect in the brain does raise the possibility that the effect of the drug on viability arises due to the effect of the drug in other tissues; however, while we cannot formally rule out this possibility, it seems likely that the effects of these drugs on Cdk5-associated survival are occurring due to their effects in the brain, since this is the tissue where Cdk5 is active and where Cdk5 pathology is observed. Importantly, this discordance also hints at a potential explanation for why some drugs, for example, antioxidants, fail to deliver beneficial effects in preclinical and clinical trials: the effect of a drug *in vivo* may cause the opposite of its intended effect in the target tissue (Forman and Zhang, 2021; Halliwell, 2024).

These data make clear that altered Cdk5 activity influences the response to redox-altering drugs. Cdk5 plays numerous physiological roles in neuronal physiology and previous transcriptional analyses have linked mitochondria and mitochondrial redox changes to the neurodegenerative consequences of changes in Cdk5 activity (Spurrier et al., 2018). Yet, existing phosphoproteomics data do not yield a mechanistic link between Cdk5 and mitochondrial redox biology (Contreras-Vallejos et al., 2014; Wen et al., 2014). Existing data do, however, offer some potential avenues for further exploration. Sun and colleagues have shown that the H_2_O_2_-reducing peroxiredoxins Prx1 and Prx2 are direct targets of Cdk5-mediated inhibition in mouse brain, which is consistent with our results showing that reducing Cdk5 activity decreases mitochondrial H_2_O_2_ (Sun et al., 2008). Further data suggests that Cdk5 regulates the mitochondrial fission regulator Drp1, which may alter the mitochondrial redox state secondary to alterations in the balance of mitochondrial fusion and fission (Jahani-Asl et al., 2015; Ježek and Plecitá-Hlavatá, 2009). While it remains unclear why reducing Cdk5 activity alters responses to redox-altering drugs, the existing data as well as the data presented here offer a functional connection between Cdk5 and mitochondrial biology that may prove useful in dissecting the underlying mechanistic links in the future.

The data presented here have implications for both how we think about ROS in disease processes and how we approach ROS as a target for therapeutics. In recent years, it has become clear that the effects of ROS cannot be considered in a monolithic way. Above, we have found that whether altering a reactive oxygen species *in vivo* is beneficial or detrimental depends on the identity of the molecule in question and the age and genotype of the individual. Additionally, whether a particular redox-altering drug could act as a beneficial therapeutic depends on those same variables and also on the age at treatment and the duration of treatment. Furthermore, the net outcome of a drug effect for the individual can be due to the direct effect of the drug and/or the compensatory mechanism(s) it activates. These data underscore the need to separate the many influences of cell and tissue biology on ROS and of ROS on that biology to better understand pathogenesis and develop more effective therapeutics.

## MATERIALS AND METHODS

### Fly maintenance, stocks, and aging

All flies were housed at 25°C and 50% humidity on a 12:12 h light:dark cycle. Unless otherwise specified, flies were fed a standard cornmeal-molasses *Drosophila* media (Caltech media; KD Medical, Columbia, MD, USA). All experiments were performed in male adults in an Oregon Red (w^+^) background. The stocks used in these experiments were as follows: *201Y-GAL4* (BDSC, #4440), *UAS-mito-roGFP2-Grx1* (BDSC, #67664), *UAS-mito-roGFP2-Orp1* (BDSC, #67667), and *UAS-mCD8-GFP* (BDSC, #5130). The Cdk5α-KO condition (*w^+^; Cdk5α ^20C^/Df(Cdk5α)^C2^; +*) has been described in detail elsewhere (Connell-Crowley et al., 2000; 2007; Spurrier et al., 2018; Trunova and Giniger, 2012).

For assessment of the correlation of mitochondrial superoxide levels in the whole brain compared to the mushroom bodies, the MB γ-neuron specific *GAL4* driver *201Y-GAL4* was used to express *UAS-mCD8-GFP* in a WT genetic background (*w^+^; 201Y-GAL4/+; UAS-mCD8-GFP/+*). For the assessment of mitochondrial glutathione redox potential, the MB γ-neuron specific *GAL4* driver *201Y-GAL4* was used to express *UAS-mito-roGFP2-Grx1* in WT and Cdk5α-KO conditions. The resulting experimental genotypes were: WT (*w^+^; 201Y-GAL4/UAS-mito-roGFP2-Grx1; +*) and Cdk5α-KO (*w^+^; Cdk5α2°C, 201Y-GAL4/Df(Cdk5α)^C2^, UAS-mito-roGFP2-Grx1; +*). For the assessment of mitochondrial H_2_O_2_ level, *201Y-GAL4* was used to express *UAS-mito-roGFP2-Orp1* in WT and Cdk5α-KO conditions. The resulting experimental genotypes were: WT (*w^+^; 201Y-GAL4/UAS-mito-roGFP2-Orp1; +*) and Cdk5α-KO (*w^+^; Cdk5α2°C, 201Y-GAL4/Df(Cdk5α)^C2^, UAS-mito-roGFP2-Orp1; +*). For all other experiments, the experimental genotypes used were: WT (*w^+^; +; +*) and Cdk5α-KO (*w^+^; Cdk5α ^20C^/Df(Cdk5α)^C2^; +*).

For aging of adult flies, males and females were collected within 24 h of eclosion and transferred to fresh vials. After 3 days, males were separated and placed into fresh vials. Flies were aged until they reached the appropriate experimental age. Flies were flipped to fresh vials twice weekly while aging.

In previous studies, *GAL4*-driven expression of *UAS-nls-mCherry* was used to quantify MB neurons (Spurrier et al., 2018). However, control experiments revealed that chronic treatment with the drugs used here produced variable effects on the efficiency of expression and detection of the fluorescent reporter, precluding the use of this method in the current study.

### Acute and chronic redox drug feeding

Drug stocks were prepared by dissolving each drug in an appropriate vehicle solvent (Table S1). Note that there were no within-group differences based on the vehicle used (Fig. S4). Drug stocks were aliquoted and stored at −20°C. For acute drug feeding, flies were first maintained and aged on standard cornmeal-molasses *Drosophila* media as described above for the first 9 days after eclosion. Drug stocks were then diluted with H_2_O supplemented with 5% sucrose (w/v) to the experimental concentration. A Kimwipe (Kimberly-Clark, Irving, TX, USA) was placed into an empty vial, and 1 ml of drug or vehicle solution was then added. Flies were then transferred to the vial containing drug or vehicle solution and maintained as described above for 24 h. Alternatively, for the comparative transcriptomics of acute versus chronic redox-altering drug treatment experiment, acute drug feeding was performed as described above with the following changes (to allow for direct comparison to chronic drug feeding): drug stocks were diluted with H_2_O to the experimental concentration before drug or vehicle media was prepared by mixing equal volumes of drug or vehicle solution at the experimental concentration with Formula 4-24 Instant *Drosophila* Medium (Carolina Biological Supply Company, Burlington, NC, USA). For chronic drug feeding, drug stocks were first diluted with H_2_O to the experimental concentration. Drug or vehicle media was then prepared by mixing equal volumes of drug or vehicle solution at the experimental concentration and Formula 4-24 Instant *Drosophila* Medium. Flies were then transferred to drug or vehicle media and maintained and aged as described above with flipping to fresh drug or vehicle media twice weekly while aging.

### General confocal microscopy and image processing

All images were acquired using a LSM 880 confocal microscope (Zeiss, Oberkochen, Germany). All images for a given experiment used identical imaging settings. Note that because of differences in imaging settings between experiments, raw values differ between acute and chronic feeding experiments and these raw values should not be compared between experiments. All image analysis was done using ImageJ (version 1.53t, NIH, Bethesda, MD, USA). All image visualizations were done using ImageJ and Imaris software (version 9.5, Oxford Instruments, Abingdon, UK). Z-stack images were visualized in Imaris using the 3D View tool. Images were rotated and cropped to remove out-of-plane signal as necessary.

### Mitochondrial superoxide level assessment

To stain brains with MitoSox-Red, brains were first dissected in 1× PBS before incubating with 30 µM MitoSox-Red (Thermo Fisher Scientific, Washington, DC, USA; #M36008) in 1× PBS for 10 min at room temperature protected from light. Brains were then washed twice with 1× PBS for 2 min. Brains were mounted on slides between two spacers (number 1 glass coverslips, to prevent squishing of brains) and covered with Vectashield mounting medium (Vector Laboratories, Newark, CA, USA) and a coverslip.

Brains were imaged immediately after dissection and incubation using a 10× (0.45 NA) air objective to acquire a Z-stack with a 4.0 µm step size covering approximately 120 µm. The laser settings were excitation at 561 nm and emission between 567-625 nm. To calculate the relative level of mitochondrial superoxide, all images were first converted to 32-bit format and thresholded with values below the threshold set to ‘Not a Number’. A ROI was then drawn around the entire brain, and the total intensity and area of the pixels with numeric values was calculated through the Z-stack. All images were then normalized to the average background fluorescence. It should be noted that MitoSox-Red also can respond to other ROS besides superoxide (Zielonka and Kalyanaraman, 2010).

To assess the correlation of mitochondrial superoxide levels in the whole brain compared to the mushroom bodies, 10-day-old WT flies expressing *201Y>mCD8-GFP* were stained with MitoSox-Red and imaged as described above with the addition of a laser line for the GFP channel with excitation at 488 nm and emission between 491-560 nm. The MitoSox-Red intensity was calculated for the whole brain as described above. The MitoSox-Red intensity was also calculated for the mushroom bodies in the same way as for the whole brain except that the ROI was drawn around the mushroom bodies (using the GFP signal as a mask) instead of around the whole brain. The whole brain and mushroom bodies MitoSox-Red intensity values from the same brain were then plotted to find the correlation between MitoSox-Red intensity values derived from each region.

### mtDNA copy number quantification

For isolation of DNA for mtDNA copy number quantification, groups of ∼20 male flies were collected by flash freezing in liquid nitrogen. Fly heads were recovered by vortexing to separate heads from bodies and subsequently passing the heads and bodies through two sieves (with the upper sieve retaining the bodies and the lower sieve retaining the heads). DNA was extracted from groups of heads using DNAzol Reagent (Thermo Fisher Scientific; #10503027) according to manufacturer's instructions. Quantification of mtDNA copy number was performed in triplicate by qPCR amplification of the mitochondrial gene *mt:CoI* and the nuclear gene *Rpl32* (as an endogenous control) using SYBR green reagents (Thermo Fisher Scientific; #4309155). The following primers were used: *mt:CoI* (forward: 5′-GATTAGGACATCCTGGAGC-3′; reverse: 5′-GCACTAATCAATTTCCAAATCC-3′) and *Rpl32* (forward: 5′-AAGCGGCGACGCACTCTGTT-3′; reverse: 5′-GCCCAGCATACAGGCCCAAG-3′). The qPCR reaction was performed on a QuantStudio 6 Flex system (Thermo Fisher Scientific; #4485691) using standard conditions: 50°C for 2 min; 95°C for 2 min; and 40 cycles of 95°C for 15 s and 60°C for 1 min. The mtDNA copy number was calculated using the 2^−ΔΔCT^ method with 10-day-old WT values as the reference value.

### Cytosolic ROS level assessment

To stain brains with H_2_DCFDA, brains were first dissected in 1× PBS before incubating with 5 µM H_2_DCFDA (Thermo Fisher Scientific; #D399) for 10 min at room temperature protected from light. Brains were then thrice washed with 1× PBS for 1 min. Brains were mounted on slides between two spacers (number 1 glass coverslips, to prevent squishing of brains) and covered with Vectashield mounting medium (Vector Laboratories, Newark, CA, USA) and a coverslip.

Brains were imaged immediately after dissection and incubation using a 10× (0.45 NA) air objective to acquire a Z-stack with a 4.0 µm step size covering approximately 120 µm. The laser settings were excitation at 488 nm and emission between 490-560 nm. To calculate the relative level of cytosolic ROS, all images were first converted to 32-bit format and thresholded with values below the threshold set to ‘Not a Number’. A ROI was then drawn around the entire brain, and the total intensity and area of the pixels with numeric values was calculated through the Z-stack.

### Mitochondrial glutathione redox potential and H_2_O_2_ level assessment

To determine the dynamic range of the *201Y>mito-roGFP2-Grx1* or *201Y>mito-roGFP2-Orp1* biosensors in the *Drosophila* mushroom body, 10 day-old WT brains were dissected in 1× PBS supplemented with either 2 mM diamide (DA) or 20 mM dithiothreitol (DTT) to oxidize or reduce the biosensors, respectively. Brains were then incubated in DA or DTT for an additional 20 min. Brains were then incubated in 30 mM *N-*Ethylmaleimide (NEM) in 1× PBS for 20 min at room temperature in order to stabilize the redox-sensitive fluorophore and protect against fixation-induced thiol oxidation (Albrecht et al., 2011). Afterwards, brains were washed in 1× PBS for 5 min followed by fixation in 4% paraformaldehyde for 1 h at room temperature. Brains were then washed three times in 1× PBS for 10 min. Finally, brains were mounted on slides between two spacers (number 1 glass coverslips, to prevent squishing of brains) and covered with Vectashield mounting medium (Vector Laboratories) and a coverslip.

To assess changes in the biosensors in the context of Cdk5α-KO or drug administration, the above procedure was followed with the following changes: brains were dissected in 30 mM NEM in 1× PBS, omitting the DA or DTT in the dissection and incubation.

Each MB hemisphere was imaged using a 40× (1.2 NA) water objective to acquire a Z-stack with a 1.0 µm step size covering approximately 120 µm. The laser settings were as follows: 405 nm channel (ex: 405 nm and em: 500-550 nm), 488 nm channel (excitation at 488 nm and emission between 500-550 nm). To calculate the oxidation/reduction ratio of mitochondrial glutathione redox potential and mitochondrial H_2_O_2_ level (from *201Y>mito-roGFP2-Grx1* or *201Y>mito-roGFP2-Orp1*, respectively), all images were first converted to 32-bit format and thresholded with values below the threshold set to ‘Not a Number’. The 405 nm channel was then divided by the 488 nm channel pixel-by-pixel to create a 405 nm/488 nm ratiometric image. A ROI was then drawn around the mushroom body on this ratiometric image, and the total intensity and area of the pixels with numeric values was calculated through the Z-stack. The reported 405 nm/488 nm ratio is the average intensity per area of the ratiometric image. Note that this ratiometric measurement corrects for any differences in the absolute expression level of these biosensors.

### Survival assay

To assay survival, flies were chronically fed drug or vehicle media and aged as described above. When flies were flipped to fresh vials, the number of dead and censored male flies were counted. Any fly that escaped the vial, became stuck to the wall or plug of the vial, or became stuck to the food was censored. After 30 days, any flies that remained alive were counted.

### Transcriptomic analysis

For isolation of RNA from 10-day-old WT flies fed drug or vehicle either acutely or chronically, groups of 20 male flies were collected without the use of CO_2_ sedation by flash freezing in liquid nitrogen. Fly heads were recovered by vortexing to separate heads from bodies and subsequently passing the heads and bodies through two sieves (with the upper sieve retaining the bodies and the lower sieve retaining the heads). Groups of heads were then homogenized in Lysis Binding Mix (from MagMAX *mir*Vana Total RNA Isolation Kit; Thermo Fisher Scientific; #A27828) using Navy Eppendorf RNA Lysis Kits (Next Advance, Troy, NY, USA; #NAVYE5-RNA) and a Bullet Blender Storm 24 (Next Advance; #4116-BBY24 M). RNA was then isolated from these homogenates using the MagMAX *mir*Vana Total RNA Isolation Kit per the manufacturer's instructions in conjunction with the KingFisher Apex Purification System with 96 Deep-Well head (Thermo Fisher Scientific; #5400930).

For RNA sequencing, the quality of RNA was first assessed by TapeStation 2200 (Agilent Technologies, Santa Clara, CA, USA) and NanoDrop (Thermo Fisher Scientific). cDNA libraries were generated from 500 ng of total RNA using NEBNext Ultra II Directional Poly-A RNA Library Prep Kit for Illumina (New England Biolabs, Ipswich, MA, USA; #E7760). cDNA library quality and quantity were assessed by TapeStation 2200 and Qubit (Thermo Fisher Scientific). cDNA libraries were sequenced on a NextSeq 2000 machine using NextSeq 2000 P3 reagents (Illumina, San Diego, CA, USA).

For analysis of sequencing data, FASTQ files were first quality inspected using the FastQC (available at: https://www.bioinformatics.babraham.ac.uk/projects/fastqc/) and MultiQC (Ewels et al., 2016) tools. For reference mapping (BDGP6), the nf-core RNA-Seq Seq pipeline (version 3.10.1; available at https://nf-co.re/rnaseq) was used in conjunction with select parameters (--outFilterMultimapNmax 500, --winAnchorMultimapNmax 500, --clip_r1 12, --clip_r2 12). To enumerate counts for known genes, the featureCounts tool was used (Liao et al., 2014). To enumerate counts for transposable elements (TEs), the TEcount tool was used (Jin et al., 2015). These counts were then organized in matrix form, with features in rows and samples in columns. Features observed to not have at least one sample with a value greater than zero were filter removed. This filtered matrix was then imported into R, the matrix of counts was pedestalled by 2, log2 transformed, then cross-sample normalized. For cross-sample normalization, cyclic LOESS was applied using the normalizeBetweenArrays function available as part of the limma package. Post normalization, outliers were detected by covariance-based principal component analysis scatterplot and removed. Cross-sample normalization was repeated using transformed counts for surviving samples then noise modeled by experiment condition (CV∼mean) using the LOWESS function. Resulting fits were visually inspected and the noise cut-off for the data defined to equal the lowest mean value where the linear relationship between CV and mean was grossly lost (1.75). Features not having at least one sample value greater than the noise cut-off value were discarded with any value less floored to equal the noise cut-off value. Features were also discarded if they were observed to have a CV greater than the average CV observed at the noise cut-off value (80%). To identify differential expressed features for each possible pairwise comparison of experiment conditions, the ANOVA test under Benjamini-Hochberg, false discovery rate, and multiple comparison correction conditions was used followed by post-hoc testing using the TukeyHSD test. Features observed to have an ANOVA corrected *P*<0.05, a post-hoc *P*<0.05, and an absolute linear fold change of means>|1.5| for a comparison were deemed to be differentially expressed for the comparison, respectively. Post testing, sample-to-sample relationships were inspected by covariance-based PCA scatterplot and correlation-based heatmap using the union set of differential features across all comparisons. Differential analysis was then repeated using ANCOVA in place of ANOVA and general linear hypothesis testing in place of Tukey HSD while adjusting for drug (DMSO, 2-AAPA, mitoQ) in one pass and adjusting for drug schedule (acute, chronic) in a second, separate pass.

All sequencing data is available on GEO: GSE274587.

### Drug uptake assay

To compare uptake of different vehicle and drug solutions, male flies were maintained and aged on standard cornmeal-molasses *Drosophila* media as described above for the first 10 days after eclosion. Flies were starved for four hours in a vial containing a Kimwipe wet with 1 ml of H_2_O. Meanwhile, drugs and corresponding vehicles were diluted to experimental concentration with H_2_O supplemented with 5% sucrose (w/v) and 2.5% FD&C Blue #1 (w/v; Sigma-Aldrich, St. Louis, MO, USA; #861146) before 1 ml of this solution was added to a vial containing a Kimwipe. Approximately 10 male flies were transferred to the vial containing drug or vehicle solution and maintained as described above for 24 h. After 24 h, flies were collected and then washed with 50% ethanol, rinsed once with PBS, and ground using a mortar and pestle in fresh PBS. The supernatant was collected and the absorbance at 650 nm measured using a BioPhotometer Plus (Eppendorf, Hamburg, Germany).

### Statistical analyses

All statistical analyses were performed using Prism (GraphPad, version 10, Boston, MA, USA). Specific statistical tests are specified in the Figure Legends. Data were tested for normality using the Kolmogorov–Smirnov test and for equality of variances using the *F*-test of equality of variances or Bartlett's test. Outliers were detected using the ROUT method with Q=1% (Motulsky and Brown, 2006). All analyses were completed blind to genotype and age. One biological replicate is represented by a single fly, unless otherwise specified. When appropriate, measurements from each MB hemisphere from a single brain were treated as technical replicates, and these measurements were averaged together. All graphs show mean±s.e.m. unless otherwise noted in the figure legends.

## Supplementary Material

10.1242/biolopen.060515_sup1 Supplementary information

Table S4. Data presented in this study. Summary data presented in this study, excluding raw RNA sequencing data (see Methods).
