# Supplementary material for: Manipulating mitochondrial reactive oxygen species alters survival in unexpected ways in a Drosophila Cdk5 model of neurodegeneration
Source: Biol Open. 2024 Oct 14;13(10):bio060515. doi: 10.1242/bio.060515 (PMC11552616; doi:10.1242/bio.060515)
Supplement: Supplementary information [file biolopen-13-060515-s1.pdf]

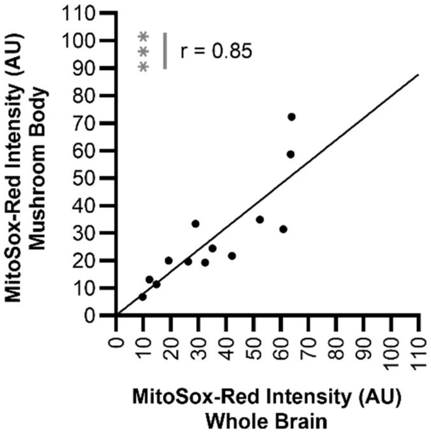

Fig. S1. MitoSox-Red intensity in the whole brain is significantly correlated with MitoSox-Red intensity in the mushroom body.

Quantification and correlation of mitochondrial superoxide level in the same 10 day-old WT (*201Y>mCD8-GFP*) brains and mushroom bodies fed standard *Drosophila* media. A higher MitoSox-Red intensity indicates a higher mitochondrial superoxide level. Pearson’s correlation coefficient was calculated between the MitoSox-Red intensity values obtained from the whole brain or mushroom bodies of the same brain. *p* values: * < .05; ** < .01, *** < .001, **** < .0001. N = 13.

Biology Open • Supplementary information

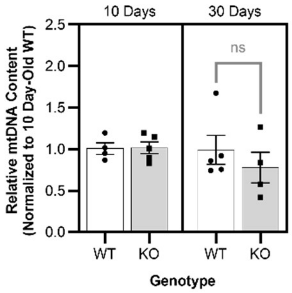

Fig. S2. Relative mtDNA copy number is not significantly different in 10 day-old or 30 day-old WT and Cdk5 α-KO heads.

Quantification of relative mtDNA copy number by qPCR (relative to a nuclear gene for normalization) in heads of WT or Cdk5α-KO flies fed standard *Drosophila* media. mtDNA copy number in all groups is normalized to that of 10 day-old WT, with ~20 male fly heads per replicate sample. Groups were compared using two-way ANOVA with Šidák's multiple comparison test. *p* values: * < .05; ** < .01, *** < .001, **** < .0001. N = 4 (WT, 10 days old), 5 (KO, 10 days old), 5 (WT, 30 days old), 4 (KO, 30 days old).

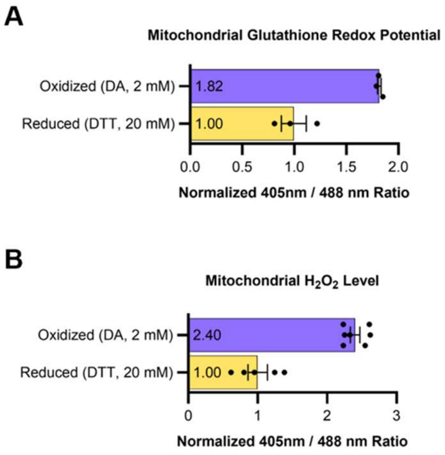

Fig. S3. mito-roGFP2 biosensors capture redox changes in the *Drosophila* mushroom body.

(A-B) Oxidation (with DA) or reduction (with DTT) of 10 day-old WT flies expressing *201Y>mito-roGFP2-Grx1* (A) or *201Y>mito-roGFP2-Orp1* (B) measuring the mitochondrial glutathione redox potential or the H_2O_2 level, respectively. 405nm/488nm ratios have been normalized by setting the average reduced 405nm/488nm ratio to 1. A higher 405nm/488nm ratio indicates a more oxidized mitochondrial glutathione redox potential (A) or a higher mitochondrial H_2O_2 level (B). N = 3 (oxidized, mitochondrial glutathione redox potential), 3 (reduced, mitochondrial glutathione redox potential), 7 (oxidized, mitochondrial H_2O_2 level), 5 (reduced, mitochondrial H_2O_2 level).

Biology Open • Supplementary information

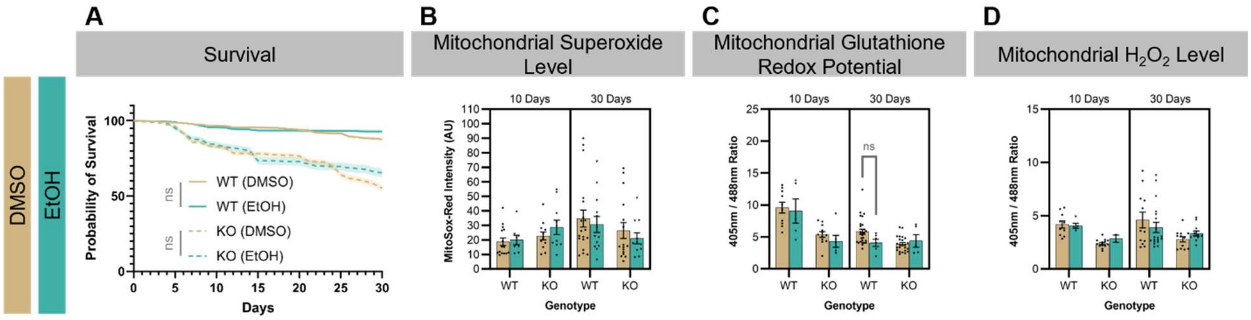

Fig. S4. WT flies fed two different vehicles, DMSO or EtOH, do not show differences in survival nor mitochondrial redox parameters.

(A) Quantification of survival in WT and Cdk5α-KO flies chronically fed vehicle (DMSO [0.1% w/v] or EtOH [0.1% w/v]). Note that these data are the same as shown in Fig. 3B-H, separated by the vehicle used for chronic feeding. Shaded background represents SEM. Differences in survival among groups were assessed by Mantel-Cox log-rank test with Bonferroni multiple comparison test. *p* values: * < .05; ** < .01, *** < .001, **** < .0001. N = 1050 (WT, DMSO), 432 (WT, EtOH), 708 (KO, DMSO), 314 (KO, EtOH).

(B) Quantification of mitochondrial superoxide level in WT or Cdk5α-KO brains chronically fed vehicle (DMSO [0.1% w/v] or EtOH [0.1% w/v]). Note that these data are the same as shown in Fig. 1B and Fig. 3B-H, separated by the vehicle used for chronic feeding. A higher MitoSox-Red intensity indicates a higher mitochondrial superoxide level. Groups were compared using two-way ANOVA with Šidák's multiple comparison test. *p* values: * < .05; ** < .01, *** < .001, **** < .0001. N = 15 (WT, 10 days old, DMSO), 8 (WT, 10 days old, EtOH), 12 (KO, 10 days old, DMSO), 10 (KO, 10 days old, EtOH), 19 (WT, 30 days old, DMSO), 13 (WT, 30 days old, EtOH), 16 (KO, 30 days old, DMSO), 11 (KO, 30 days old, EtOH).

(C) Quantification of mitochondrial glutathione redox potential in WT or Cdk5α-KO MBs chronically fed vehicle (DMSO [0.1% w/v] or EtOH [0.1% w/v]). Note that these data are the same as shown in Fig. 1B and Fig. 3B-H, separated by the vehicle used for chronic feeding. A larger 405nm/488nm ratio indicates a more oxidized mitochondrial glutathione redox potential. Groups were compared using two-way ANOVA with Šidák's multiple comparison test. *p* values: * < .05; ** < .01, *** < .001, **** < .0001. N = 10 (WT, 10 days old, DMSO), 5 (WT, 10 days old, EtOH), 12 (KO, 10 days old, DMSO), 6 (KO, 10 days old, EtOH), 27 (WT, 30 days old, DMSO), 6 (WT, 30 days old, EtOH), 24 (KO, 30 days old, DMSO), 5 (KO, 30 days old, EtOH).

(D) Quantification of mitochondrial H_2O_2 level in WT or Cdk5α-KO MBs chronically fed vehicle (DMSO [0.1% w/v] or EtOH [0.1% w/v]). Note that these data are the same as shown in Fig. 1B and Fig. 3B-H, separated by the vehicle used for chronic feeding. A larger 405nm/488nm ratio indicates a higher mitochondrial H_2O_2

level. Groups were compared using two-way ANOVA with Šidák's multiple comparison test. *p* values: * < .05; ** < .01, *** < .001, **** < .0001. N = 10 (WT, 10 days old, DMSO), 5 (WT, 10 days old, EtOH) , 13 (KO, 10 days old, DMSO), 2 (KO, 10 days old, EtOH), 12 (WT, 30 days old, DMSO), 19 (WT, 30 days old, EtOH), 12 (KO, 30 days old, DMSO), 11 (KO, 30 days old, EtOH).

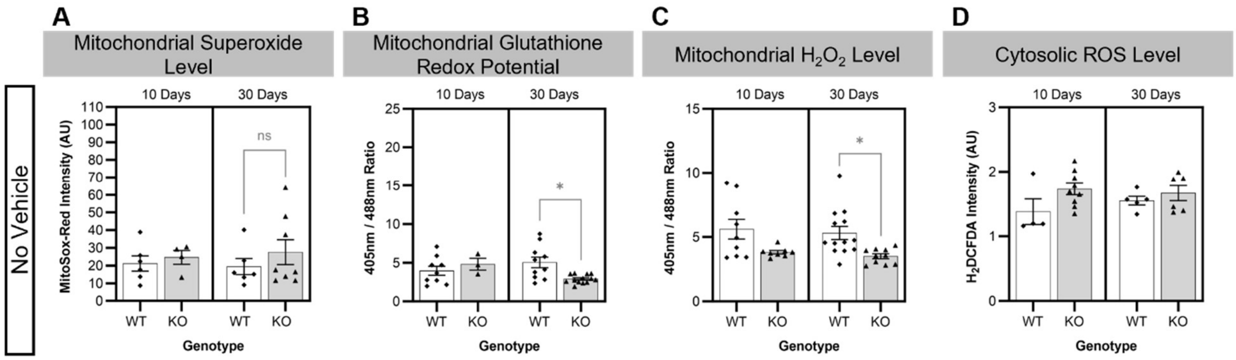

Fig. S5. Mitochondrial and cytosolic ROS in WT and Cdk5α-KO flies raised on standard *Drosophila* media and not exposed to vehicle.

(A) Quantification of mitochondrial superoxide level in WT or Cdk5α-KO brains. A higher MitoSox-Red intensity indicates a higher mitochondrial superoxide level. Groups were compared using two-way ANOVA with Šidák's multiple comparison test. *p* values: * < .05; ** < .01, *** < .001, **** < .0001. N = 6 (WT, 10 days old), 4 (KO, 10 days old), 6 (WT, 30 days old), 8 (KO, 30 days old).

(B) Quantification of mitochondrial glutathione redox potential in WT or Cdk5α-KO MBs. A larger 405nm/488nm ratio indicates a more oxidized mitochondrial glutathione redox potential. Groups were compared using two-way ANOVA with Šidák's multiple comparison test. *p* values: * < .05; ** < .01, *** < .001, **** < .0001. N = 9 (WT, 10 days old), 3 (KO, 10 days old), 10 (WT, 30 days old), 13 (KO, 30 days old). (C) Quantification of mitochondrial H_2O_2 level in WT or Cdk5α-KO MBs. A larger 405nm/488nm ratio indicates a higher mitochondrial H_2O_2 level. Groups were compared using two-way ANOVA with Šidák's multiple comparison test. *p* values: * < .05; ** < .01, *** < .001, **** < .0001. N = 9 (WT, 10 days old), 8 (KO, 10 days old), 13 (WT, 30 days old), 10 (KO, 30 days old).

(D) Quantification of normalized H_2DCFDA in WT or Cdk5α-KO brains. A higher H_2DCFDA intensity indicates a higher cytosolic ROS level. Groups were compared using two-way ANOVA with Šidák's multiple comparison test. *p* values: * < .05; ** < .01, *** < .001, **** < .0001. N = 4 (WT, 10 days old), 9 (KO, 10 days old), 5 (WT, 30 days old), 6 (KO, 30 days old).

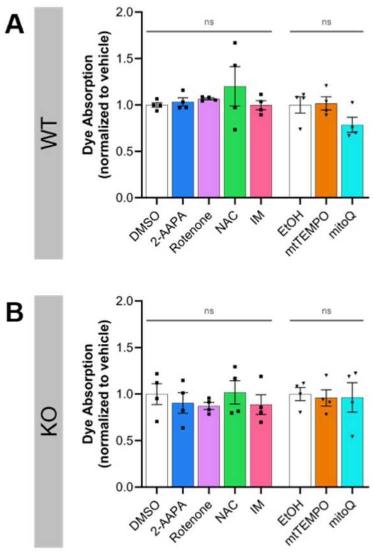

Fig. S6. Redox altering drugs do not alter the uptake of food relative to vehicle control in WT and KO flies.

Absorbance at 650 nm of FD&C Blue #1 added to drug- or vehicle-containing food and consumed by groups of 10 day-old WT (A) or KO (B) flies over 24 hours. Absorbance normalized to vehicle controls (either DMSO or EtOH). DMSO-containing groups (DMSO, 2-AAPA, rotenone, NAC, and IM) and EtOH-containing groups (EtOH, mtTEMPO, and mitoQ) were compared using Kruskal-Wallis tests with Dunn's multiple comparison tests. *p* values: * < .05; ** < .01, *** < .001, **** < .0001. N = 4 (all groups).

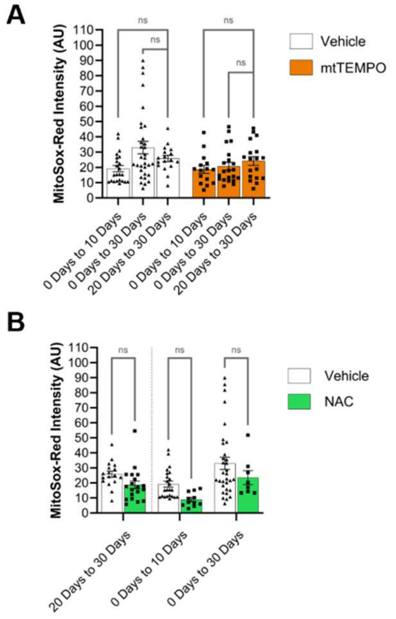

Fig. S7. Effects of redox-altering drug treatment duration vs. age at treatment assessment.

(A) Comparison of mitochondrial superoxide level among flies fed mtTEMPO or among flies fed vehicle from 20 days old until 30 days old. Data are the same as that presented in Figure 4B. Data from quantification of mitochondrial superoxide level of flies fed mtTEMPO from 0 days old until either 10 days old or 30 days old are the same as that presented in Figure 3. Vehicle-treated groups and mtTEMPO-treated groups were compared using one-way ANOVAs with Dunnett's multiple comparison tests. *p* values:

* < .05; ** < .01, *** < .001, **** < .0001. N = 17 (vehicle, 20 days to 30 days), 18 (mtTEMPO, 20 days to 30 days), 23 (vehicle, 0 days to 10 days), 15 (mtTEMPO, 0 days to 10 days), 32 (vehicle, 0 days to 30 days), 21 (mtTEMPO, 0 days to 30 days).

(B) Comparison of mitochondrial superoxide level in flies fed NAC *vs.* vehicle from 20 days old until 30 days old. Data are the same as that presented in Figure 4C. Data from quantification of mitochondrial superoxide level of flies fed NAC from 0 days old until either 10 days old or 30 days old are the same as that presented in Figure 3. Vehicle-treated groups were compared to NAC-treated groups for each chronic feeding paradigm by two-way ANOVA with Šidák's multiple comparison test. *p* values: * < .05; ** < .01, *** < .001, **** < .0001. N = 23 (vehicle, 0 days to 10 days), 32 (vehicle, 0 days to 30 days), 17 (vehicle, 20 days to 30 days), 11 (NAC, 0 days to 10 days), 8 (NAC, 0 days to 30 days), 19 (NAC, 20 days to 30 days).

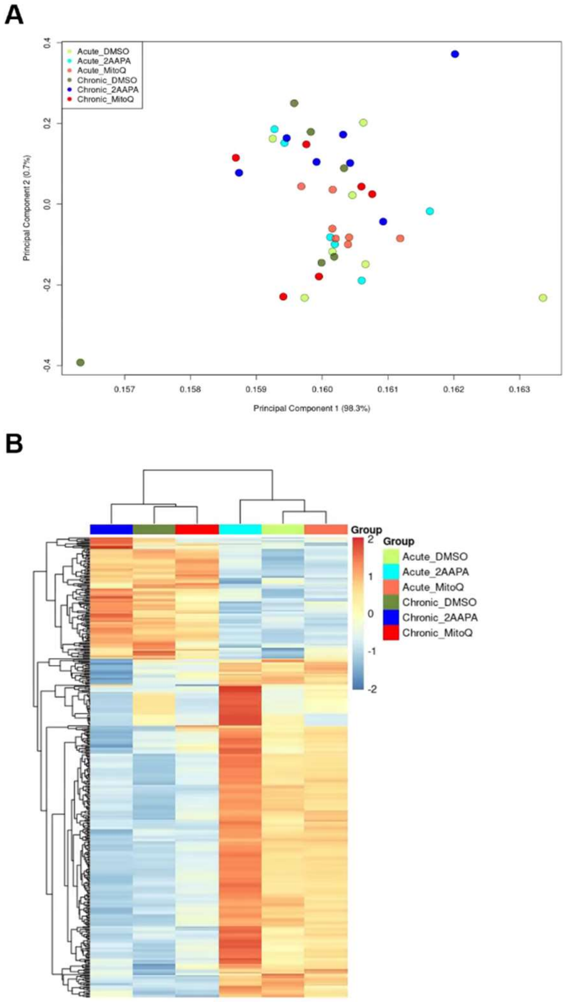

Fig. S8. Transcriptomic profiling of acute and chronically fed 10 day-old WT flies reveals changes associated with vehicle treatment between acute and chronic groups, but not among drug treatments.

(A) PCA plot of 10,597 genes surviving noise cutoff from 10 day-old WT flies fed either DMSO (vehicle), 2-AAPA, or mitoQ acutely or chronically. N = 7 (acute, DMSO), 6 (acute, 2-AAPA), 7 (acute, mitoQ), 6 (chronic, DMSO), 7 (chronic, 2-AAPA), 6 (chronic, mitoQ).

(B) Gene heatmap of 184 differentially expressed genes that contribute significantly to the variance amongst samples. Selected genes had a linear fold change of means > |1.5| and a corrected $p < .05$. N = 7 (acute, DMSO), 6 (acute, 2-AAPA), 7 (acute, mitoQ), 6 (chronic, DMSO), 7 (chronic, 2-AAPA), 6 (chronic, mitoQ).

Table S1. Summary of drugs used in this study.

Drugs used in this study with CAS iden fica on number, supplier informa on, solvents and concentra ons used to prepare drug for feeding. For non-standard drug abbrevia ons, a full drug name is included if one exists. Drugs and their dosages were selected based on literature search for drugs and their dosages that have been shown to exert an effect in *Drosophila* without inducing overt toxicity.

Drug Abbreviation	Drug Name	CAS Number	Supplier	Catalog Number	Vehicle Solvent	Drug Concentration
**2-AAPA**		1133387-90-2	Sigma-Aldrich	A4111	DMSO	10 µM
**3-AT**	3-amino-1,2,4-triazole	61-82-5	Sigma-Aldrich	A8056	H2O	500 µM
**Antimycin A**		1397-94-0	Sigma-Aldrich	A8674	EtOH	9.11 µM
**Auranofin**		34031-32-8	Sigma-Aldrich	A6733	DMSO	1 µM
**BSO**	Buthionine sulfoximine	83730-53-4	Cayman Chemical	14484	H2O	10 mM
**DDC**	Diethyldithiocarbamate	20624-25-3	Sigma-Aldrich	D3506	H2O	1 mM
**EUK-8**		53177-12-1	Sigma-Aldrich	SML0742	DMSO	500 µM
**Idebenone**		58186-27-9	Sigma-Aldrich	I5659	DMSO	10 µM
**IM**	Imisopasem manganese	218791-21-0	MedChemExpress	HY-13336	DMSO	500 µM
**mitoQ**	Mitoquinone mesylate	845959-50-4	Cayman Chemical	29317	EtOH	100 µM
**mtTEMPO**	Mito-TEMPO	1334850-99-5	Sigma-Aldrich	SML0737	EtOH	1 mM
**NAC**	N-acetyl cysteine	616-91-1	Sigma-Aldrich	A7250	DMSO	1 mM
**PQ**	Paraquat	1910-42-5	Sigma-Aldrich	36541	H2O	100 µM
**Rotenone**		83-79-4	Sigma-Aldrich	R8875	DMSO	10 µM
**S1QEL1.1**		897613-29-5	Cayman Chemical	20982	DMSO	800 nM
**S3QEL2**		890888-12-7	Cayman Chemical	18556	DMSO	800 nM

Table S2. Summary of drugs used in acute drug administration screen and their predicted effects.

Drugs used in acute drug administration screen with their mechanisms of action and their predicted effects on survival, mitochondrial superoxide level, mitochondrial glutathione redox potential, and mitochondrial H_2O_2 level. The predicted drug effects are based on their mechanisms of action as well as experimental evidence in various *in vitro* and *in vivo* contexts. The predicted drug effects are indicated by the green up arrow (drug predicted to improve survival), the magenta down arrow (drug predicted to reduce survival), the orange up arrow (drug predicted to lead to an increased oxidation state), the blue down arrow (drug predicted to lead to a decreased oxidation state), or a black bar (drug predicted to led to no change in survival or oxidation state or a prediction cannot be made for the given drug). References for predicted effects based on observations in model systems are indicated by a superscripted numeral (*e.g.*, [1]), and references are included in Supplementary References. Predicted effects that are based only on the mechanism of action of a drug are indicated by a superscripted pound ([#]).

Drug	Target of Action	Effect on Target	Survival	Superoxide	Glutathione	H_2O_2
**2-AAPA**	TrxR & GR	Inhibitor	↓[1]	–	↑[2]	–
**3-AT**	Catalase	Inhibitor	↓[3]	–	–	↑[4]
**Antimycin A**	ETC complex III	Inhibitor	↓[5]	↑[6]	↑[7]	↑[7]
**Auranofin**	TrxR	Inhibitor	↓[#]	–	↑[8]	–
**BSO**	GCL	Inhibitor	↓[9]	–	↓[8]	–
**DDC**	Cu/Zn-SOD	Inhibitor	↑[10]	↑[11]	↓[10]	↓[12]
**EUK 8**	Mn-SOD & catalase	Mimetic	↑[13]	↓[#]	–	↑[#]
**Idebenone**	CoQ(10)	Mimetic	↑[14]	↓[#]	–	↓[#]
**IM**	Mn-SOD	Mimetic	↑[15]	↓[15]	–	↑[#]
**mitoQ**	CoQ(10)	Mimetic	↑[16]	↓[17]	–	↓[18]
**mtTEMPO**	Free radicals	Scavenger	↑[19]	↓[20]	–	–
**NAC**	Glutathione	Precursor	↑[21]	–	–	↑[7]
**Paraquat**	Free radicals	Toxicant	↓[22]	↑[23]	↑[24]	↑[25]
**Rotenone**	ETC complex I	Inhibitor	↓[26]	↑[27]	↑[28]	↑[29]
**S1QEL1.1**	ETC complex I	Suppressor of electron leak	↑[#]	↓[#]	–	↓[30]
**S3QEL2**	ETC complex III	Suppressor of electron leak	↑[#]	↓[31]	–	↓[31]

Key	
↑ (green)	**Improves** survival
↓ (magenta)	**Reduces** survival
↑ (orange)	**Increases** oxidation state
↓ (blue)	**Decreases** oxidation state
–	**No change**

Table S3. Sample sizes for Fig. 3C-H. Sample sizes for data shown in Fig. 3C-H.

		Vehicle	2-AAPA	Rotenone	NAC	IM	mtTEMPO	mitoQ
**Survival**								
	**WT**	1482	556	551	609	464	509	443
	**KO**	1022	412	388	451	364	370	348
**Mitochondrial Superoxide Level**								
**10 Day-Old**	**WT**	23	11	13	11	12	15	13
	**KO**	22	9	9	5	11	11	10
**30 Day-Old**	**WT**	32	21	13	8	18	21	15
	**KO**	27	10	8	1	10	12	12
**Mitochondrial Glutathione Redox Potential**								
**10 Day-Old**	**WT**	15	7	9	8	7	8	9
	**KO**	18	6	5	8	6	9	9
**30 Day-Old**	**WT**	33	9	7	11	7	6	8
	**KO**	29	9	12	9	11	8	7
**Mitochondrial H_2O_2 Level**								
**10 Day-Old**	**WT**	15	7	7	6	9	9	6
	**KO**	15	8	8	7	7	7	9
**30 Day-Old**	**WT**	31	10	8	6	9	8	9
	**KO**	23	4	7	4	6	6	5

Table S4. Data presented in this study.

Summary data presented in this study, excluding raw RNA sequencing data (see Methods).

Available for download at
https://journals.biologists.com/bio/article-lookup/doi/10.1242/bio.060515#supplementary-data

Biology Open • Supplementary information

SUPPLEMENTARY REFERENCES

1. **de Souza, L. F., Schmitz, A. E., da Silva, L. C. S., de Oliveira, K. A., Nedel, C. B., Tasca, C. I., de Bem, A. F., Farina, M., & Dafre, A. L.** (2017). Inhibition of reductase systems by 2-AAPA modulates peroxiredoxin oxidation and mitochondrial function in A172 glioblastoma cells. *Toxicology in Vitro*, *42*, 273–280. https://doi.org/10.1016/j.tiv.2017.04.028
2. **Zhao, Y., Seefeldt, T., Chen, W., Wang, X., Matthees, D., Hu, Y., & Guan, X.** (2009). Effects of glutathione reductase inhibition on cellular thiol redox state and related systems. *Archives of Biochemistry and Biophysics*, *485*(1), 56–62. https://doi.org/10.1016/j.abb.2009.03.001
3. **Nguyen, T. T., Huy, T. X. N., Aguilar, C. N. T., Reyes, A. W. B., Salad, S. A., Min, W. G., Lee, H. J., Kim, H. J., Lee, J. H., & Kim, S.** (2023). Intracellular Growth Inhibition and Host Immune Modulation of 3-Amino-1,2,4-triazole in Murine Brucellosis. *International Journal of Molecular Sciences*, *24*(24). https://doi.org/10.3390/ijms242417352
4. **Bhuyan, K. C., & Bhuyan, D. K.** (1977). Regulation of hydrogen peroxide in eye humors effect of 3-amino-1H-1,2,4-triazole on catalase and glutathione peroxidase of rabbit eye. *Biochimica et Biophysica Acta (BBA) - General Subjects*, *497*(3), 641–651. https://doi.org/10.1016/0304-4165(77)90284-7
5. **Bjedov, I., Cochemé, H. M., Foley, A., Wieser, D., Woodling, N. S., Castillo-Quan, J. I., Norvaisas, P., Lujan, C., Regan, J., Toivonen, J. M., et al.** (2020). Fine-tuning autophagy maximises lifespan and is associated with changes in mitochondrial gene expression in Drosophila. *PLOS Genetics*, *16*(11), e1009083. https://doi.org/10.1371/journal.pgen.1009083
6. **Senoo-Matsuda, N., Yasuda, K., Tsuda, M., Ohkubo, T., Yoshimura, S., Nakazawa, H., Hartman, P. S., & Ishii, N.** (2001). A Defect in the Cytochrome b Large Subunit in Complex II Causes Both Superoxide Anion Overproduction and Abnormal Energy Metabolism in Caenorhabditis elegans. *Journal of Biological Chemistry*, *276*(45), 41553–41558. https://doi.org/10.1074/jbc.M104718200
7. **Albrecht, S. C., Barata, A. G., Großhans, J., Teleman, A. A., & Dick, T. P.** (2011). In vivo mapping of hydrogen peroxide and oxidized glutathione reveals chemical and regional specificity of redox homeostasis. *Cell Metabolism*, *14*(6), 819–829. https://doi.org/10.1016/j.cmet.2011.10.010
8. **Martínez-González, J. J., Guevara-Flores, A., Rendón, J. L., & Arenal, I. P. D.** (2015). Auranofin-induced oxidative stress causes redistribution of the glutathione pool in Taenia crassiceps cysticerci. *Molecular and Biochemical Parasitology*, *201*(1), 16–25. https://doi.org/10.1016/j.molbiopara.2015.05.001

9. **Zhao, H. W., Zhou, D., & Haddad, G. G.** (2011). Antimicrobial peptides increase tolerance to oxidant stress in Drosophila melanogaster. *Journal of Biological Chemistry*, *286*(8), 6211–6218. https://doi.org/10.1074/jbc.M110.181206

10. **Sohal, R. S., Farmer, K. J., Allen, R. G., & Ragland, S. S.** (1984). Effects of diethyldithiocarbamate on life span, metabolic rate, superoxide dismutase, catalase, inorganic peroxides and glutathione in the adult male housefly, Musca domestica. *Mechanisms of Ageing and Development*, *24*(2), 175–183. https://doi.org/10.1016/0047-6374(84)90069-1

11. **Clément, M. V., & Stamenkovic, I.** (1996). Superoxide anion is a natural inhibitor of FAS-mediated cell death. *The EMBO Journal*, *15*(2), 216–225. https://doi.org/10.1002/j.1460-2075.1996.tb00352.x

12. **Petersen, A. B., Gniadecki, R., Vicanova, J., Thorn, T., & Wulf, H. C.** (2000). Hydrogen peroxide is responsible for UVA-induced DNA damage measured by alkaline comet assay in HaCaT keratinocytes. *Journal of Photochemistry and Photobiology B: Biology*, *59*(1–3), 123–131. https://doi.org/10.1016/S1011-1344(00)00149-4

13. **Melov, S., Ravenscroft, J., Malik, S., Gill, M. S., Walker, D. W., Clayton, P. E., Wallace, D. C., Malfroy, B., Doctrow, S. R., & Lithgow, G. J.** (2000). Extension of Life-Span with Superoxide Dismutase/Catalase Mimetics. *Science*, *289*(5484), 1567–1569. https://doi.org/10.1126/science.289.5484.1567

14. **Soriano, S., Llorens, J. V., Blanco-Sobero, L., Gutiérrez, L., Calap-Quintana, P., Morales, M. P., Moltó, M. D., & Martínez-Sebastián, M. J.** (2013). Deferiprone and idebenone rescue frataxin depletion phenotypes in a Drosophila model of Friedreich's ataxia. *Gene*, *521*(2), 274–281. https://doi.org/10.1016/j.gene.2013.02.049

15. **Filograna, R., Godena, V. K., Sanchez-Martinez, A., Ferrari, E., Casella, L., Beltramini, M., Bubacco, L., Whitworth, A. J., & Bisaglia, M.** (2016). Superoxide Dismutase (SOD)-mimetic M40403 is protective in cell and fly models of paraquat toxicity: Implications for Parkinson disease. *Journal of Biological Chemistry*, *291*(17), 9257–9267. https://doi.org/10.1074/jbc.M115.708057

16. **Ng, L. F., Gruber, J., Cheah, I. K., Goo, C. K., Cheong, W. F., Shui, G., Sit, K. P., Wenk, M. R., & Halliwell, B.** (2014). The mitochondria-targeted antioxidant MitoQ extends lifespan and improves healthspan of a transgenic Caenorhabditis elegans model of Alzheimer disease. *Free Radical Biology and Medicine*, *71*, 390–401. https://doi.org/10.1016/j.freeradbiomed.2014.03.003

17. **Santini, E., Turner, K. L., Ramaraj, A. B., Murphy, M. P., Klann, E., & Kaphzan, H.** (2015). Mitochondrial Superoxide Contributes to Hippocampal Synaptic Dysfunction and Memory Deficits

in Angelman Syndrome Model Mice. *The Journal of Neuroscience*, *35*(49), 16213–16220. https://doi.org/10.1523/JNEUROSCI.2246-15.2015

18. **Huang, W., Cash, N., Wen, L., Szatmary, P., Mukherjee, R., Armstrong, J., Chvanov, M., Tepikin, A. V., Murphy, M. P., Sutton, R., & Criddle, D. N.** (2015). Effects of the mitochondria-targeted antioxidant mitoquinone in murine acute pancreatitis. *Mediators of Inflammation*, *2015*. https://doi.org/10.1155/2015/901780

19. **Rimal, S., Tantray, I., Li, Y., Pal Khaket, T., Li, Y., Bhurtel, S., Li, W., Zeng, C., & Lu, B.** (2023). Reverse electron transfer is activated during aging and contributes to aging and age-related disease. *EMBO Reports*, *24*(4). https://doi.org/10.15252/embr.202255548

20. **Yang, S. G., Park, H. J., Kim, J. W., Jung, J. M., Kim, M. J., Jegal, H. G., Kim, I. S., Kang, M. J., Wee, G., Yang, H. Y., et al.** (2018). Mito-TEMPO improves development competence by reducing superoxide in preimplantation porcine embryos. *Scientific Reports*, *8*(1). https://doi.org/10.1038/s41598-018-28497-5

21. **Shaposhnikov, M. V., Zemskaya, N. V., Koval, L. A., Schegoleva, E. V., Zhavoronkov, A., & Moskalev, A. A.** (2018). Effects of N-acetyl-L-cysteine on lifespan, locomotor activity and stress-resistance of 3 Drosophila species with different lifespans. *Aging*, *10*(9), 2428–2458. https://doi.org/10.18632/aging.101561

22. **Vermeulen, C. J., Van De Zande, L., & Bijlsma, R.** (2005). Resistance to oxidative stress induced by paraquat correlates well with both decreased and increased lifespan in Drosophila melanogaster. *Biogerontology*, *6*(6), 387–395. https://doi.org/10.1007/s10522-005-4903-2

23. **Farrington, J. A., Ebert, M., Land, E. J., & Fletcher, K.** (1973). Bipyridylium quaternary salts and related compounds. V. Pulse radiolysis studies of the reaction of paraquat radical with oxygen. Implications for the mode of action of bipyridyl herbicides. *Biochimica et Biophysica Acta (BBA) - Bioenergetics*, *314*(3), 372–381. https://doi.org/10.1016/0005-2728(73)90121-7

24. **Brigelius, R., Dostal, L. A., Horton, J. K., & Bend, J. R.** (1986). Alteration of the Redox State of Nadph and Glutathione in Perfused Rabbit Lung By Paraquat. *Toxicology and Industrial Health*, *2*(4), 417–428. https://doi.org/10.1177/074823378600200405

25. **Castello, P. R., Drechsel, D. A., & Patel, M.** (2007). Mitochondria are a major source of paraquat-induced reactive oxygen species production in the brain. *Journal of Biological Chemistry*, *282*(19), 14186–14193. https://doi.org/10.1074/jbc.M700827200

26. **Ayajuddin, M., Phom, L., Koza, Z., Modi, P., Das, A., Chaurasia, R., Thepa, A., Jamir, N., Neikha, K., & Yenisetti, S. C.** (2022). Adult health and transition stage-specific rotenone-mediated

Drosophila model of Parkinson's disease: Impact on late-onset neurodegenerative disease models. *Frontiers in Molecular Neuroscience*, *15*. https://doi.org/10.3389/fnmol.2022.896183

27. **Turrens, J. F., & Boveris, A.** (1980). Generation of superoxide anion by the NADH dehydrogenase of bovine heart mitochondria. *Biochemical Journal*, *191*(2), 421–427. https://doi.org/10.1042/bj1910421

28. **Molina-Jiménez, M. F., Sánchez-Reus, M. I., Andres, D., Cascales, M., & Benedi, J.** (2004). Neuroprotective effect of fraxetin and myricetin against rotenone-induced apoptosis in neuroblastoma cells. *Brain Research*, *1009*(1–2), 9–16. https://doi.org/10.1016/j.brainres.2004.02.065

29. **Lambert, A. J., & Brand, M. D.** (2004). Inhibitors of the quinone-binding site allow rapid superoxide production from mitochondrial NADH:ubiquinone oxidoreductase (complex I). *Journal of Biological Chemistry*, *279*(38), 39414–39420. https://doi.org/10.1074/jbc.M406576200

30. **Brand, M. D., Goncalves, R. L. S., Orr, A. L., Vargas, L., Gerencser, A. A., Borch Jensen, M., Wang, Y. T., Melov, S., Turk, C. N., Matzen, J. T., et al.** (2016). Suppressors of Superoxide-H2O2 Production at Site IQ of Mitochondrial Complex I Protect against Stem Cell Hyperplasia and Ischemia-Reperfusion Injury. *Cell Metabolism*, *24*(4), 582–592. https://doi.org/10.1016/j.cmet.2016.08.012

31. **Morris, O., Deng, H., Tam, C., & Jasper, H.** (2020). Warburg-like Metabolic Reprogramming in Aging Intestinal Stem Cells Contributes to Tissue Hyperplasia. *Cell Reports*, *33*(8). https://doi.org/10.1016/j.celrep.2020.108423

Biology Open • Supplementary information
